# Association of urate-lowering therapies with abdominal aortic aneurysm growth and clinical events in men: A population-based cohort study

**DOI:** 10.1371/journal.pone.0341242

**Published:** 2026-07-31

**Authors:** Katja Bøhling Dybdahl, Mathilde Bernal, Marie Dahl, DANCAVAS trialists, Lasse M. Obel, Jes Sanddal Lindholt, Joachim S. Skovbo

**Affiliations:** 1 Faculty of Health Sciences, University of Southern Denmark, Odense, Denmark; 2 Department of Cardiothoracic and Vascular Surgery, Odense University Hospital, Odense, Denmark; 3 Department of Clinical Research, University of Southern Denmark, Odense, Denmark; 4 Vascular Research Unit, Department of Vascular Surgery, Viborg Regional Hospital, Viborg, Denmark; 5 Department of Clinical Medicine, Aarhus University, Aarhus, Denmark; 6 Department of Cardiology, Odense University Hospital, Odense, Denmark; 7 Department of Cardiology, Little Belt Hospital, Vejle, Denmark; 8 Department of Cardiology, Diagnostic Centre, Regional Hospital Silkeborg, Silkeborg, Denmark; 9 Department of Medicine, Odense University Hospital, Svendborg, Denmark; 10 Department of Clinical Biochemistry, Odense University Hospital, Odense, Denmark; 11 Elite Centre for Individualized Medicine in Arterial Disease (CIMA), Odense University Hospital, Odense, Denmark; Calico: Calico Life Sciences LLC, UNITED STATES OF AMERICA

## Abstract

**Objective:**

Abdominal aortic aneurysms (AAA) are potentially life-threatening if they rupture. Xanthine oxidase inhibitors have been suggested to reduce aneurysm growth via urate-lowering and antioxidative mechanisms, though their clinical efficacy remains uncertain.

The primary objective of this prospective cohort study was to estimate potential associations between urate-lowering therapies and AAA growth, and secondarily risk of rupture, surgery, all-cause mortality, and major adverse cardiovascular events (MACE), in men aged 65–74.

**Design:**

This population-based cohort study included data from two large cardiovascular screening trials.

**Methods:**

The two Danish AAA male cohorts contributed with baseline characteristics, including AAA measurements through ultrasound and computed tomography scans. Participants were followed for five years through linkage with the Danish national health registries. Prescription records defined the exposure of urate-lowering therapies, and participants were categorised as users or non-users. Growth rate was examined using multivariate linear regression, and the secondary outcomes were evaluated using Cox regression.

**Results:**

This study included 998 men, of whom 73 (7.3%) were exposed to urate-lowering therapies, with a mean defined daily dose of 0.377 per day. During 3 993 person-years, an insignificant mean difference of 0.28 mm/year (95%CI: −0.96–1.52, p = .66) in AAA growth was observed between users and non-users. No associations between urate-lowering therapies and risk of surgery, rupture, all-cause mortality, or MACE were observed.

**Conclusion:**

The use of urate-lowering therapies was not associated with a reduction in AAA progression. These findings do not support a clinically meaningful effect of urate-lowering therapies on aneurysm progression in men with AAA.

## Introduction

Abdominal aortic aneurysms (AAA) are highly lethal, often remaining silent until rupture, which is associated with a high mortality rate [1]. Advanced age and male sex are well-established risk factors, with men over 65 years being particularly exposed [2].

One hypothesis of AAA development is that oxidative stress contributes through chronic inflammation and vascular damage [3,4]. Xanthine oxidase, an enzyme involved in generating reactive oxygen species, is present in aneurysmal tissue and is a source of uric acid [5,6]. Hyperuricemia has been linked to aneurysm formation and progression in genetic and clinical studies [7–9]. Urate-lowering therapies (ULT), such as allopurinol and febuxostat, inhibit xanthine oxidase and reduce serum urate.

Despite extensive research, no pharmacologic agent has been definitively shown to attenuate aneurysm growth, leaving surgical intervention as the only established option [10]. Drug repurposing offers a promising avenue, given existing medications’ established safety profiles and lower development costs. While metformin and statins show promise, conclusive data supporting their efficacy are still awaited, and further studies are ongoing [11,12]. Alternatively, ULT may reduce oxidative stress and thereby theoretically slow aneurysm progression, however, their impact on AAA growth has not been clearly established [4,13].

Due to shared risk profiles with AAA, e.g., age, male sex, hypertension, and smoking, major adverse cardiovascular events (MACE) represent another critical outcome, which includes acute myocardial infarction, stroke, and all-cause mortality [14–18]. Hyperuricemia has been associated with MACE, however, the impact of ULT on MACE remains controversial, with conflicting findings [19–22].

This cohort study’s main objective was to evaluate the potential effect of ULT on AAA growth in men aged 65–74 over a five-year follow-up. Secondarily, the study sought to estimate associations of ULT with i) AAA rupture, ii) need for surgical intervention, iii) all-cause mortality, and iv) MACE.

## Methods

### Study design

This cohort study utilised prospectively collected data from two large Danish population-based screening trials. Reporting followed the Strengthening the Reporting of Observational Studies in Epidemiology guidelines (STROBE) [23].

### Materials

Viborg Vascular Screening Trial (VIVA) was a randomised, clinically controlled screening trial in the Central Region of Denmark, enrolling 50 165 men aged 65–74 between 2008 and 2011 [24].

The Danish Cardiovascular Screening Trial (DANCAVAS) was a multicentre, randomised, controlled interventional screening trial in the Southern Region of Denmark, enrolling 46 611 men aged 65–74 from 2014 to 2018 [25]. Both studies provided baseline measurements and characteristics, along with follow-up imaging.

Follow-up data were obtained from the Danish national registries and linked via unique personal identification numbers, ensuring complete follow-up:

i) Data on the use of urate-lowering therapies (ULT) and other medications were extracted from the Danish National Prescription Registry using the Anatomical Therapeutic Chemical Classification System. The registry includes information on drug dosage, quantity, and redeemed prescriptions from all pharmacies in Denmark dating back to 1994 [26].
ii) The Danish National Patient Register, with the International Statistical Classification of Diseases and Related Health Problems, 10^th^ revision [27], captured ruptured AAA, surgery, and MACE events.
iii) Information on cause-specific mortality was obtained from the Danish Cause of Death Registry [28], which records primary and contributing causes of death using the International Statistical Classification of Diseases and Related Health Problems, 10^th^ revision.
iv) Finally, the Danish Education Registry identified each individual’s highest educational level [29].

### Participants

The study population comprised all men in VIVA and DANCAVAS who received a diagnosis of AAA, defined as an aortic diameter ≥30 mm, by ultrasound or computed tomography, respectively. Participants with AAA ≥ 55 mm were excluded, as they were promptly referred for surgical intervention.

### Imaging and surveillance protocols

In VIVA, trained nurses in mobile clinics used portable ultrasound to measure the abdominal aortic diameter at peak systole, applying an inner-to-inner anterior–posterior approach. Participants were followed with annual scans until the diameter exceeded 55 mm [24].

In the DANCAVAS trial, non-contrast electrocardiogram-gated computed tomography was used to assess the abdominal aorta, measuring the maximal outer-to-outer anterior-posterior diameter perpendicular to the aortic axis. Specific scanners and settings used at the five screening sites have been detailed elsewhere [14,30]. Follow-up intervals were based on aneurysm diameter, with imaging twice a year for 30–44 mm, annual imaging for 45–49 mm, and semi-annual imaging for 50–54 mm diameters. [25].

### Follow-up

The screening date marked the index date, initiating up to five years of follow-up or until aneurysm rupture, surgery, death, or end of data on December 31, 2021. Follow-up was registry-based.

### Outcomes and exposure

During follow-up, participants were classified as unexposed until the first prescription was redeemed. Exposure status was assigned only after the redemption of a second prescription, to minimise misclassification due to the risk of early discontinuation.

The term ULT refers to both allopurinol and febuxostat unless otherwise specified.

AAA growth rate was defined as the change in maximal abdominal aortic diameter, measured in millimetres, from baseline to the last measurement. Ruptured AAA was defined by hospital diagnosis or rupture as the cause of death. A surgical AAA event was defined as the first procedure, either open or endovascular aneurysm repair.

MACE was defined as a modified three-point outcome including AMI, stroke, and all-cause mortality [31]. All-cause mortality was substituted for cardiovascular death, as the cause of death was often based on clinical judgment without autopsy [32]. For a complete list of variable definitions, see Table in S1 File.

### Statistical analysis

#### Baseline characteristics.

Categorical variables were summarised as counts and percentages, while continuous variables were described using means and standard deviations for normally distributed data or medians with interquartile ranges (IQR) for non-normally distributed data. Group differences were evaluated using the Wilcoxon Rank-Sum test for numerical variables and Pearson’s Chi-squared test for categorical variables. Baseline medications were defined by ≥1 prescriptions redeemed up to three months before the individual’s index date.

### AAA growth rates

**Fig 1:**
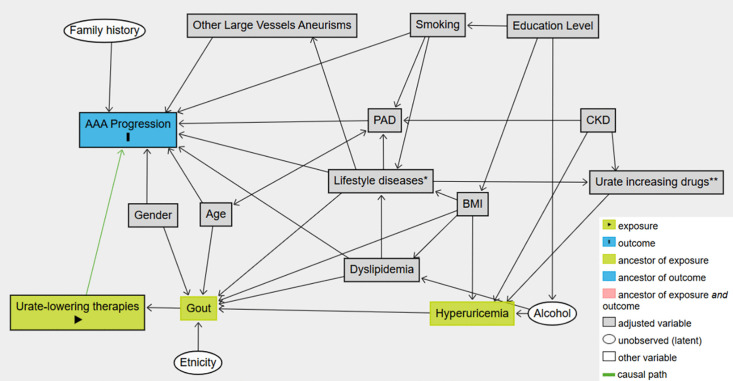
Directed acyclic graph displaying key associations and potential confounders affecting the relationship between urate-lowering therapies use and AAA progression [49,50]. AAA = Abdominal aortic aneurysm, BMI = Body mass index, CKD = Chronic kidney disease, PAD = Peripheral artery disease. *Includes diabetes, hypertension, chronic obstructive lung disease, and cardiovascular disease. **Includes beta blockers, thiazides and loop-diuretics.

Growth rates for each participant were determined using linear regression, incorporating all available measurements, reducing the influence of variability between each measurement. Linear regression was selected due to the linear pattern observed in the growth rates. The calculated growth rate was then used in subsequent analysis as a continuous variable. The multivariable regressions were adjusted for the sub study, that includes the image modality and other well-established clinically and physiologically risk factors, including baseline age, baseline aorta size, body mass index (BMI), smoking status, diastolic blood pressure, diabetes, chronic obstructive pulmonary disease (COPD), chronic kidney disease, peripheral arterial disease, presence of other large vessel aneurysms, educational level and use of metformin, statins and urate-increasing drugs, including thiazides, loop diuretics and beta-blockers (Fig 1) [14,33]. Participants with only a single abdominal aortic scan did not contribute to growth rate analyses, as at least two scans were required to assess aneurysm progression.

The primary analysis compares AAA growth with the average daily use of ULT in defined daily dose (DDD) throughout the follow-up period. This method of averaged DDD per day was chosen to account for varying follow-up times in the linear regression. From a clinical perspective, 1 DDD equals 400 mg for allopurinol and 80 mg for febuxostat.

### Secondary outcomes

Incidence rates per 1 000 person-years were calculated for AAA rupture, risk of needing AAA surgery, all-cause mortality, and MACE, respectively. Unadjusted and adjusted Cox regression analyses were performed to assess the hazard ratios of ULT on these outcomes. To account for competing risks, composite endpoints were analysed, including rupture or surgery, and a broader composite of rupture, surgery, or death. The multivariable regressions were adjusted for the same covariates as those included in the primary outcome analyses. Given their relevance to clinical eligibility for surgery at the time, unoperated lung cancer and heart failure were also included in the adjusted models by the expert review of the senior author (JL).

The proportional hazards assumption was assessed globally and for each covariate using scaled Schoenfeld residuals. Where deviations were observed, sensitivity analyses with time-varying coefficients were performed to confirm the robustness of the primary estimates.

To mitigate immortal time bias in the event-based analyses, a long-format data approach was utilised, measuring cumulative exposure of ULT in increments of 1 000 DDD. This method ensured that cumulative exposure increased progressively, accurately reflecting the level of exposure at each time point.

Analyses were performed in Stata 18 with a 5% significance level [34].

### Power calculation

Sample size calculations indicated that the least detectable difference with 73 observed users in a merged cohort of 998 men would be 0.814 mm/year aneurysm growth over 5 years, based on knowledge of a mean growth of 2.21 mm standard deviation of 2.395 mm of two merged cohorts, a two-sided α of 0.05, and 80% power [35]. A clinically significant reduction has earlier been estimated to be at 1 mm/year, as trials of drugs like doxycycline and metformin have aimed for at least 1 mm/year difference versus placebo [36,37]. Consequently, the power of the study is sufficient to clarify whether ULT could be useful for clinical use to impair.

### Ethics

The VIVA study was approved by the Ethics Committee of Central Denmark Region (journal number: M-20080028, see text in S2 File) and the Danish Data Protection Agency (journal number: 1-16-02-1-08). The DANCAVAS trials were approved by the Southern Denmark Region Committee on Biomedical Research Ethics (journal number: S-20140028, see text in S3 File and S4 File) and the Danish Data Protection Agency. All participants gave informed consent. Data from Statistics Denmark were anonymous, with counts under three (excluding zero) reported as ‘< 3’ to protect participant privacy per regulations [38].

## Results

### Participants characteristics

**Fig 2:**
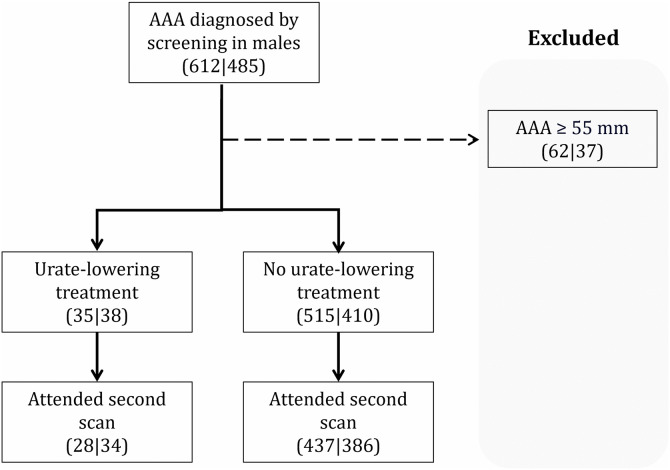
Flowchart illustrating participant inclusion from the VIVA and DANCAVAS screening trials. Numbers are presented as VIVA|DANCAVAS. VIVA = The Viborg Vascular Screening Trial. DANCAVAS = The Danish Cardiovascular Screening Trial. AAA = Abdominal aortic aneurysms.

The study population included 998 male participants with AAA from the VIVA (n = 550) and DANCAVAS (n = 448) cohorts, with a median age of 69.5 years (IQR: 67;72). During the follow-up period, 35 VIVA and 38 DANCAVAS participants received urate-lowering therapies (ULT), with febuxostat administered to three participants in total. The mean DDD of ULT among users prior to the index date was 0.205 DDD/day (Fig 2, Table 1).

**Table 1:** Characterisation of the two Danish population-based male AAA cohorts from the VIVA and DANCAVAS trials according to use of urate-lowering therapies.

Variables	Total	Non-users	Users	P-value
**N (VIVA|DANCAVAS)**	998 (550|448)	925 (515|410)	73 (35|38)	
**Age, years**	69.5 (67; 72)	69 (67; 72)	70 (67; 71)	.91
**Size of AAA at baseline, mm**	37.2 ± 6.28	37.2 ± 6.26	37.4 ± 6.52	.92
**Body mass index, kg/m** ^**2**^	28.2 ± 4.31	28 ± 4.08	30.9 ± 5.9	**<.001**
**ULT prior to index date, DDD/day**	0 (0; 0)	0 (0; 0)	0.205 (0; 0.41)	**<.001**
**Systolic blood pressure, mmHg**	152 ± 20.6	153 ± 20.3	148 ± 24.1	.30
**Diastolic blood pressure, mmHg**	85.1 ± 11.4	85.1 ± 11.4	84.9 ± 11.4	.75
**Smoking status**				**.003**
*Never*	82 (8.22)	73 (7.89)	9 (12.3)	
*Former*	524 (52.5)	475 (51.4)	49 (67.1)	
*Current*	390 (39.1)	375 (40.5)	15 (20.5)	
**Medication at baseline**				
*Anticoagulants*	89 (8.92)	78 (8.43)	11 (15.1)	.055
*Platelet inhibitors*	459 (46)	416 (45)	43 (58.9)	**.021**
*NSAID*	249 (24.9)	223 (24.1)	26 (35.6)	**.029**
*Beta blockers*	304 (30.5)	268 (29)	36 (49.3)	**<.001**
*Thiazides*	141 (14.1)	131 (14.2)	10 (13.7)	.91
*Loop diuretics*	141 (14.1)	131 (14.2)	10 (13.7)	.91
*ACE inhibitors*	302 (30.3)	269 (29.1)	33 (45.2)	**.004**
*AT2-antagonists*	195 (19.5)	177 (19.1)	18 (24.7)	.25
*Calcium channel blockers*	305 (30.6)	281 (30.4)	24 (32.9)	.66
*Broncho dilators*	150 (15)	132 (14.3)	18 (24.7)	**.017**
*Metformin*	88 (8.82)	71 (7.68)	17 (23.3)	**<.001**
*Insulin*	23 (2.3)	18 (1.95)	5 (6.85)	**.007**
Other oral antidiabetics*	107 (10.7)	85 (9.19)	22 (30.1)	**<.001**
*Statins*	579 (58)	526 (56.9)	53 (72.6)	**.009**
*Urate-increasing drugs* ^*§*^	379 (38)	340 (36.8)	39 (53.4)	**.005**
*Urate-decreasing drugs* ^*¶*^	423 (42.4)	388 (41.9)	35 (47.9)	.32
**Co-morbidities**				
*Hypertension*	666 (66.7)	603 (65.2)	63 (86.3)	**<.001**
*Dyslipidemia*	635 (63.6)	577 (62.4)	58 (79.5)	**.004**
*Diabetes*	122 (12.2)	98 (10.6)	24 (32.9)	**<.001**
*Chronic obstructive lung disease*	223 (22.3)	197 (21.3)	26 (35.6)	**.005**
*Chronic kidney disease*	29 (2.91)	24 (2.59)	5 (6.85)	**.037**
*Peripheral arterial disease*	220 (22)	208 (22.5)	12 (16.4)	.23
*Other large vessel aneurysms*	151 (15.1)	133 (14.4)	18 (24.7)	**.018**
*Heart failure*	78 (7.82)	64 (6.92)	14 (19.2)	**<.001**
*Alcohol induced liver disease*	5 (.501)	5 (.541)	0 (0)	.53
*Major adverse cardiovascular event* ^*‡*^	263 (26.4)	241 (26.1)	22 (30.1)	.45
*Myocardial infarction*	182 (18.2)	163 (17.6)	19 (26)	.073
*Stroke*	101 (10.1)	96 (10.4)	5 (6.85)	.34

Categorical variables are presented as n (%), continuous variables are presented as means with ± standard deviation (SD) if normally distributed. Otherwise, they are reported as medians with (interquartile range) (IQR). The Wilcoxon Rank-Sum test and Pearson’s Chi2 test for numerical and categorical variables, respectively. All medicine data is prescriptions one year prior to inclusion. AAA = Abdominal aortic aneurysm. VIVA = The Viborg Vascular Screening Trial. DANCAVAS = The Danish Cardiovascular Screening Trial. ULT = Urate-lowering therapies. DDD = Defined daily dose. NSAID = Non-steroidal anti-inflammatory drug. ACE-inhibitor = Angiotensin converting enzyme inhibitor. AT2-antagonist = Angiotensin-II-receptor antagonist. MACE = Major adverse cardiovascular event. ^*^Do not include metformin. ^§^Beta-blockers, thiazides and loop-diuretics. ^¶^AT2-antagonists and calcium channel blockers. ^‡^Includes acute myocardial infarction, stroke and all-cause mortality.

### Descriptive data

Users of ULT had a higher burden of comorbidities and greater medication use compared with non-users. Statistically significant differences were observed in BMI, hypertension, diabetes, dyslipidemia, COPD, other large-vessel aneurysms, chronic kidney disease, and heart failure. Urate-increasing drugs were more common among users of ULT who were also more likely to be former smokers, whereas non-users had a higher prevalence of current smoking (Table 1).

The mean exposure of ULT was low, 0.377 DDD/day, while the mean cumulative exposure of ULT during follow-up was 419 DDD. Due to 11 (15.1%) non-attenders among exposed participants at the second scan, 62 (84.9%) exposed participants contributed to the growth rate analysis in the user group (Table 2).

**Table 2:** Dose-dependent outcomes of urate-lowering therapies in AAA cohorts from the VIVA and DANCAVAS screening trials.

Variables	Total	Non-users	Users
**N (VIVA|DANCAVAS)**	998 (550|448)	925 (515|410)	73 (35|38)
**Urate-lowering therapies, DDD/Day**	0.028 ± 0.118	0 ± 0	0.377 ± 0.245
**Urate-lowering therapies (cumulative), DDD**	0 (0; 0)	0 (0; 0)	419 (229; 725)
**Multiple AAA scans**	823 (82.5)	761 (82.3)	62 (84.9)
**Follow-up time, years**	4.97 (3.2; 5)	4.95 (3.19; 5)	4.97 (3.55; 5)
**Growth rate, mm/year**	2.16 ± 2.38	2.19 ± 2.39	1.77 ± 2.29
**Surgery**	212 (21.2)	199 (21.5)	13 (17.8)
*Incidence rate*	53 [CI: 46; 61]	54 [CI: 47; 62]	43 [CI: 25; 74]
**Ruptured AAA**	15 (1.5)	15 (1.62)	0 (0)
*Incidence rate*	3.8 [CI: 2.3; 6.2]	4.1 [CI: 2.5; 6.7]	0 [CI: 0; 0]
Deaths*	132 (13.2)	120 (13)	12 (16.4)
*Incidence rate*	33 [CI: 28; 39]	33 [CI: 27; 39]	40 [CI: 22; 70]
**All-cause mortality** ^**§**^	152 (15.2)	138 (14.9)	14 (19.2)
*Incidence rate* ^*§*^	33 [CI: 28; 39]	33 [CI: 28; 39]	42 [CI: 25; 72]
**MACE** ^**§, ¶**^	203 (20.3)	187 (20.2)	16 (21.9)
*Incidence rate* ^*§*^	47 [CI: 41; 54]	46 [CI: 40; 54]	51 [CI: 31; 84]

Categorical variables are presented as n (%), continuous variables are presented as means with ± standard deviation (SD) if normally distributed. Otherwise, they are reported as medians with (interquartile range) (IQR). Statistically significant p-values are marked in bold. AAA = Abdominal aortic aneurysm. VIVA = The Viborg Vascular Screening Trial. DANCAVAS = The Danish Cardiovascular Screening Trial. DDD = Defined daily dose. Incidence rate = per 1 000 person-years. CI = 95% confidence interval. MACE = Major adverse cardiovascular event. ^*^Before surgery or ruptured AAA was experienced. ^§^Follow-up was up to given event, completion of five years follow-up, death, or 31-12-2021, whichever came first. ^¶^Includes acute myocardial infarction, stroke and all-cause mortality.

### Main results

The median overall follow-up time was 4.97 years (IQR: 3.2;5.0), corresponding to a total follow-up of 3 993 person-years. A total of 864 prescriptions were redeemed, including 11 for febuxostat. The AAA growth rate was 1.77 (±2.29) mm/year in users and 2.19 (±2.39) mm/year in non-users (Table 2). The unadjusted change in growth rate based on the average DDD/day was −0.50 mm/year (95%CI: −1.9–0.91, p = .49).

**Fig 3:**
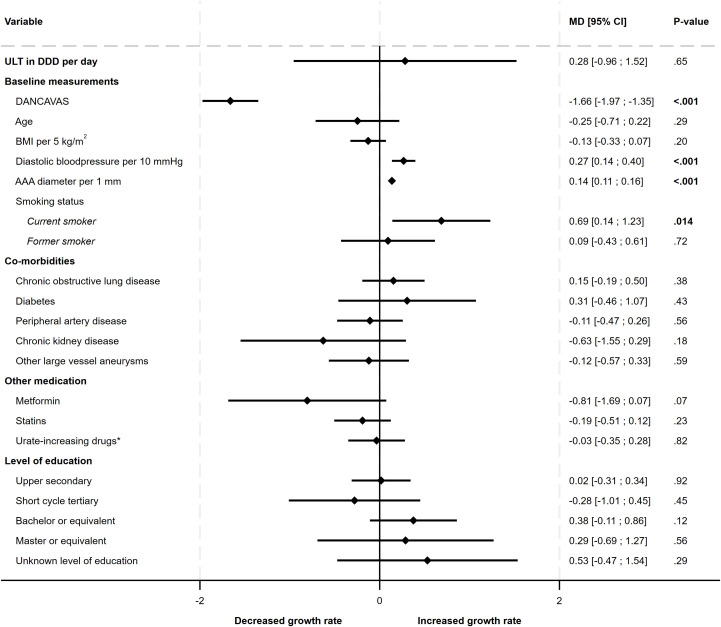
Association between average dose of urate-lowering therapies and abdominal aortic aneurysm growth: adjusted linear regression analysis using data from two population-based screening trials. ULT = Urate-lowering therapies. DDD = Defined daily dose. CI = Confidence interval. DANCAVAS = The Danish Cardiovascular Screening Trial. * Includes beta blockers, thiazides and loop-diuretics.

When adjusting for confounders, no statistically significant association between average DDD/day of ULT and AAA growth (0.8 mm/year, 95%CI: −0.96–1.52, p = .66). However, participation in the DANCAVAS study was linked to a marked reduction in AAA growth (−1.66 mm/year, 95%CI: −1.97 – −1.35, p < .001) (Fig 3).

Current smoking and diastolic blood pressure were independently associated with increased growth of 0.69 mm/year (95%CI: 0.14–1.23, p = .014) and 0.27 mm/year (95%CI: 0.14–0.40, p < .001), respectively. Diabetes as a diagnosis displayed a statistically insignificant tendency towards an increased growth rate, while metformin use was associated with decreased growth (Fig 3). Increasing age was not associated with AAA growth, and educational level showed no consistent association.

### Secondary outcomes

Aneurysm rupture occurred in zero users and 15 (1.6%) non-users. Surgical repair was performed in 13 (17.8%) users and 199 (21.5%) non-users. All-cause mortality affected 14 (19.2%) users and 138 (14.9%) non-users. Finally, MACE occurred in 16 (21.9%) users and 187 (20.2%) non-users (Table 2).

ULT showed no significant association with rupture, risk of needing surgery, all-cause mortality, or MACE in both unadjusted and adjusted models (Table 3). Composite endpoints combining surgery or rupture and surgery, rupture or death likewise indicated no associations. The proportional hazards assumption was satisfied in the analyses of all-cause mortality, MACE, and the composite of surgery and rupture. In two of the three surgery-related outcomes, global Schoenfeld tests indicated modest deviation (p between 0.01 and 0.04), attributable to baseline aortic size (time-interaction hazard ratio 0.97 per year, 95% CI 0.948 to 0.997). The deviation was no longer significant when stratified by study, consistent with heterogeneity between imaging modalities. Primary estimates were materially unchanged in sensitivity analyses including a time-varying coefficient for aortic size.

**Table 3:** Unadjusted and adjusted cox regression for secondary outcomes in the VIVA and DANCAVAS cohorts.

	Unadjusted (n = 998)			Adjusted^¶^ (n = 972)		
**Events**	**HR [95%CI]**	**P-value**	**Events**	**HR [95%CI]**	**P-value**
**Surgery**	212	1.1 [0.33; 3.6]	.88	209	1.6 [0.47; 5.7]	.44
**Surgery or rupture**	227	0.95 [0.28; 3.2]	.94	224	1.4 [0.4; 5]	.59
**Surgery, rupture, or death**	359	1.5 [0.69; 3.2]	.30	351	1.7 [0.76; 3.9]	.19
**Rupture**	15	NA	1.0	15	NA	NA
All-cause mortality*	152	2.2 [0.87; 5.4]	.095	147	1.9 [0.68; 5.5]	.21
MACE* ^**, §**^	203	1.7 [0.64; 4.7]	.28	197	1.7 [0.56; 4.9]	.36

N refers to the number of individuals included in the Cox regression. HRs are based on the cumulative dosage of urate-lowering therapies per 1 000 DDD. Statistically significant p-values are marked in bold. HR = Hazard ratio. 95%CI = 95% confidence interval. VIVA = The Viborg Vascular Screening Trial. DANCAVAS = The Danish Cardiovascular Screening Trial. DDD = Defined daily dose. MACE = Major adverse cardiac events. NA = Non-Available. *Follow-up was up to given event, completion of five years follow-up, death, or 31-12-2021, whichever came first. ^§^Includes acute myocardial infarction, stroke and all-cause mortality. ^**¶**^Adjusted for sub study, that includes the image modality, baseline age, baseline aorta size, body mass index, smoking status, diastolic blood pressure, diabetes, chronic obstructive pulmonary disease, chronic kidney disease, peripheral arterial disease, presence of other large vessel aneurysms, educational level, unoperated lung cancer, heart failure and use of metformin, statins and urate-increasing drugs, including thiazides, loop diuretics and beta-blockers.

**Fig 4:**
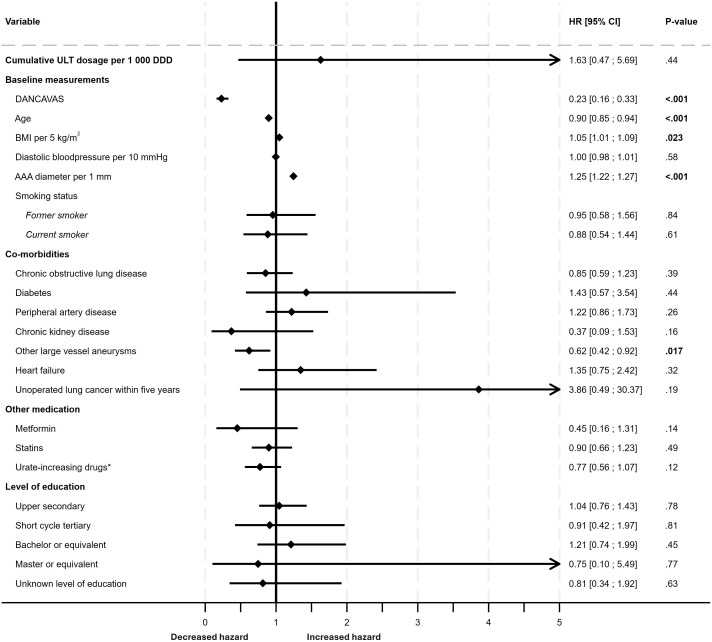
Association between cumulative dose of urate-lowering therapies and risk of needing surgery in abdominal aortic aneurysms: adjusted cox regression analysis. ULT = Urate-lowering therapies. DDD = Defined daily dose. CI = Confidence interval. DANCAVAS = The Danish Cardiovascular Screening Trial. *Includes beta blockers, thiazides and loop-diuretics.

**Fig 5:**
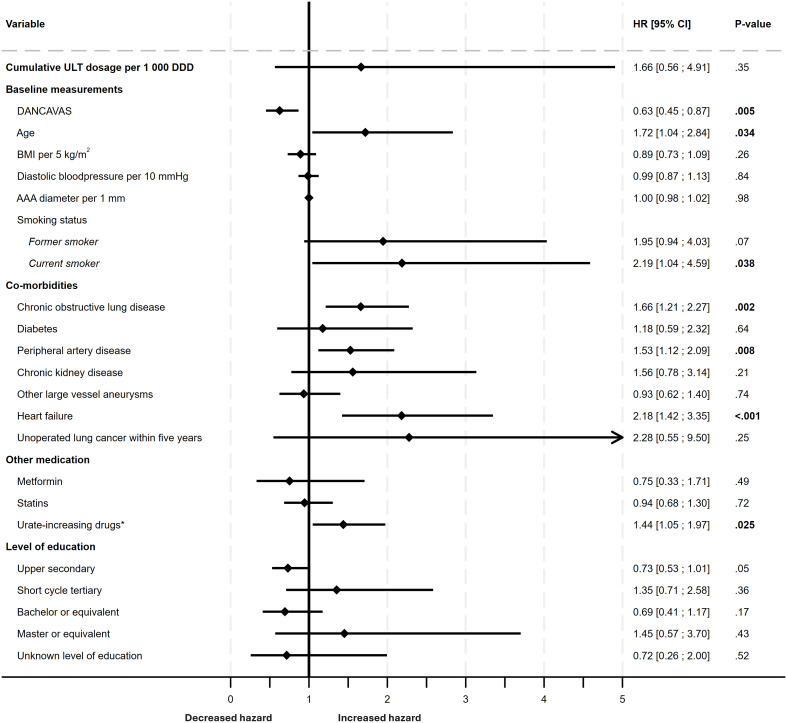
Association between cumulative dose of urate-lowering therapies and risk of major adverse cardiovascular events*: an adjusted cox regression. ULT = Urate-lowering therapies. DDD = Defined daily dose. CI = Confidence interval. DANCAVAS = The Danish Cardiovascular Screening Trial. *Includes acute myocardial infarction, stroke and all-cause mortality. **Includes beta blockers, thiazides and loop-diuretics.

After adjustment, increasing age and the presence of other large-vessel aneurysms were linked to reduced risk of needing surgery, whereas higher BMI was associated with increased risk of surgery. Notably, participation in DANCAVAS was associated with both a markedly lower risk of needing surgery and MACE (Figs 4 and 5).

Moreover, use of urate-increasing drugs was linked with a higher risk of MACE (Fig 5). Adjusted estimates were based on 972 (97.4%) participants**,** due to missing confounder data (Table 3).

## Discussion

Urate-lowering therapies (ULT), including xanthine oxidase inhibitors such as allopurinol and febuxostat, have been proposed to reduce oxidative stress and thereby potentially attenuate aneurysm progression. In this cohort study, based on data from two large population-based screening trials, no associations were observed between the use of ULT and the change in AAA growth, risk of rupture, risk of undergoing surgical repair, all-cause mortality, or MACE.

Prior studies on ULT as medication for aortic diseases are heterogeneous in design and methods, and findings have been contradictory. A preclinical study in Marfan mouse models suggested a protective effect of allopurinol on thoracic aortic disease [39], however, a recent systematic review examining the potential effects of pharmacologic agents on AAA progression did not identify ULT as promising therapeutic candidates [11]. Furthermore, a small observational study reported that allopurinol did not reduce vascular superoxide production and further suggested that xanthine oxidase is not a major contributor to oxidative stress in AAA [40].

In this study, participants with AAA receiving ULT, the rate of surgical repair was not reduced. Mortality analyses indicated that a higher rate of pre-operative death could not explain this finding. This is an important consideration, as the users of ULT had a higher burden of comorbidities and more extensive medication use.

Interestingly, the presence of other large vessel aneurysms was associated with a reduced risk of needing surgery [41]. A possible hypothesis is that these patients may have more complex aneurysm anatomy with a higher risk of repair and increased cut point of repair, usually set to 6.0 cm, or repair was considered contraindicated due to severely increased risk of repair.

A potential effect modification was observed between diabetes and metformin use concerning the risk of needing surgery. While diabetes was associated with an increased hazard for surgery, this association appeared attenuated among metformin users. This aligns with ongoing studies investigating metformin as a potential protective therapy in AAA [11,42]. However, the first of four randomised controlled trials was unable to show an association between metformin and reduced aneurysm growth [37], suggesting that metformin may function more as a marker for diabetes rather than a therapeutic agent.

Urate-increasing drugs were associated with a higher risk of MACE, potentially due to increased oxidative stress, as hypothesised in prior studies [5,6], although it is equally plausible that this reflects a higher burden of comorbidity. Importantly, the use of ULT did not result in a reduction in MACE events.

Participants from the DANCAVAS cohort demonstrated notably better outcomes regarding growth rate, risk of needing surgery, and MACE. This may reflect DANCAVAS being a more recent study compared to VIVA, suggesting potential differences in treatment, such as statin therapy [43,44]. Although both computed tomography, employed in DANCAVAS, and ultrasound, used in VIVA, have demonstrated comparable accuracy in assessing aortic diameter [30,45], ultrasound is inherently more susceptible to operator-dependent variability [45], which may have affected measurement precision and contributed to the observed differences in aneurysm growth rates.

### Strengths

A major strength of this study was the use of two large, protocolised, randomised, population-based screening trials with prospectively collected data. Linkage to validated, high-quality Danish registries allowed for robust outcome ascertainment [46,47], with cumulative dosage providing an accurate and clinically relevant estimate compared to baseline exposure alone.

### Limitations

Despite including two large population-based cohorts with sufficient power to detect a clinically significant effect, a major limitation was limited statistical power to detect smaller but potentially true associations, primarily due to a modest sample size and low ULT exposure (<1 DDD/day). Such limited exposure reduces the likelihood of observing a treatment effect.

Another important limitation concerns the absence of rupture events among ULT users, which limits the interpretation of ULT’s potential benefit on this clinically important outcome.

The lack of female participants, reflecting the original cohorts’ compositions, reduces the external validity of these findings for women. This is particularly important given that current AAA threshold does not reflect female anatomy [48], and women may exhibit more aggressive disease progression and poorer outcomes compared to men [1].

Gout was used as a surrogate marker for hyperuricemia, however, a clinical diagnosis of gout requires a prior flare, likely excluding individuals with asymptomatic hyperuricemia, leading to potential misclassification.

Serum urate levels were unavailable, although such data would have enhanced the accuracy when assessing the effect of ULT. Finally, confounding by indication cannot be ruled out, as the diagnosis of gout may be influenced by disease severity, comorbidities, prior treatment decisions, and lifestyle factors, which could also impact the outcomes assessed.

These limitations should be considered when interpreting the observed associations

### Perspectives

In a cohort of men with screening-detected AAA, ULT had no effect on AAA progression, rupture, risk of needing AAA surgery, all-cause mortality or MACE. A randomised trial is not recommended, as a clinically meaningful benefit appears unlikely.

As data on aneurysm growth emerge, inclusion in meta-analyses may improve effect estimates. Furthermore, prospective studies assessing whether ULT prevents AAA formation, rather than only limits expansion, could clarify its role in primary prevention. However, study prioritisation should depend on feasibility and cost.

An alternative strategy to attenuate oxidative stress in AAA has been suggested, focusing on the inhibition of nicotinamide adenine dinucleotide phosphate oxidase as a potentially more relevant therapeutic target [40]. Despite AAA’s complex pathogenesis challenging targeted therapy, agents like statins and metformin may offer promising treatment options as ongoing studies advance.

### Conclusion

In this population-based cohort study, urate-lowering therapies (ULT) use was not linked with a clinically significant reduction in AAA growth rate, risk of rupture, risk of needing AAA surgery, all-cause mortality or MACE over a five-year follow-up period. Although powered to detect clinically meaningful effects, smaller effects cannot be excluded and are unlikely to influence clinical practice. ULT use was, however, associated with higher MACE occurrence. These results do not support the use of ULT to limit aneurysm progression. Further studies are needed to explore alternative pharmacologic treatments, including metformin, which show promise in ongoing trials, and statins, which have shown potential in a recent publication.

## Supporting information

S1 File Definitions. Table of definitions used, origin of data, and specific codes, measurements or timeframes applied in the study. ATC = Anatomical Therapeutic Chemical, ICD10 = International Statistical Classification of Diseases and Related Health Problems 10th Revision, SKS = The Danish Medical Classification System, DANCAVAS = The Danish Cardiovascular Screening trial, VIVA = The Viborg Vascular trial.
(PDF)

S2 File VIVA ethics approval. (PDF)

S3 File DANCAVAS ethics final approval. (PDF)

S4 File DANCAVAS ethics approval. (PDF)

S5 File VIVA Study protocol. (PDF)

S6 File DANCAVAS study protocol. (PDF)

## Abbreviations

AAA: Abdominal aortic aneurysm
AMI: Acute myocardial infarction
BMI: Body mass index
COPD: chronic obstructive pulmonary disease
DANCAVAS: The Danish Cardiovascular Screening Trial
DDD: Defined daily dose
HR: Hazard ratio
IQR: Interquartile range
MACE: Major adverse cardiovascular events
STROBE: Strengthening the Reporting of Observational Studies in Epidemiology guidelines
P: P value
ULT: Urate-lowering therapies
VIVA: Viborg Vascular Screening Trial
95%CI: 95% confidence interval.

## References

[1] Golledge J, Thanigaimani S, Powell JT, Tsao PS. Pathogenesis and management of abdominal aortic aneurysm. Eur Heart J. 2023;44(29):2682–97. doi: 10.1093/eurheartj/ehad386 37387260 PMC10393073

[2] Nordon IM, Hinchliffe RJ, Loftus IM, Thompson MM. Pathophysiology and epidemiology of abdominal aortic aneurysms. Nat Rev Cardiol. 2011;8(2):92–102. doi: 10.1038/nrcardio.2010.180 21079638

[3] Qian G, Adeyanju O, Olajuyin A, Guo X. Abdominal aortic aneurysm formation with a focus on vascular smooth muscle cells. Life (Basel). 2022;12(2):191. doi: 10.3390/life12020191 35207478 PMC8880357

[4] Kazaleh M, Gioscia-Ryan R, Ailawadi G, Salmon M. Oxidative stress and the pathogenesis of aortic aneurysms. Biomedicines. 2023;12(1):3. doi: 10.3390/biomedicines12010003 38275364 PMC10813769

[5] Patetsios P, Rodino W, Wisselink W, Bryan D, Kirwin JD, Panetta TF. Identification of uric acid in aortic aneurysms and atherosclerotic artery. Ann N Y Acad Sci. 1996;800:243–5. doi: 10.1111/j.1749-6632.1996.tb33318.x 8959001

[6] Battelli MG, Polito L, Bortolotti M, Bolognesi A. Xanthine oxidoreductase-derived reactive species: physiological and pathological effects. Oxid Med Cell Longev. 2016;2016:3527579. 26823950 10.1155/2016/3527579 PMC4707389

[7] Lin Z-P, He H-Q, Aierken Y, Wu Y, Liu Y. Effect of serum uric acid on the risk of aortic aneurysm and dissection: a mendelian randomization analysis. Biochem Biophys Rep. 2024;38:101743. doi: 10.1016/j.bbrep.2024.101743 38873223 PMC11170348

[8] Chen J, Zhang X, Yao H, Pang J. Causal association between uric acid levels and the risk of aortic aneurysm and aortic dissection: a two-sample Mendelian randomization study. Nutr Metab Cardiovasc Dis. 2024;34(2):515–20. doi: 10.1016/j.numecd.2023.10.020 38161112

[9] Wang J-C, Tsai S-H, Tsai H-Y, Lin S-J, Huang P-H. Hyperuricemia exacerbates abdominal aortic aneurysm formation through the URAT1/ERK/MMP-9 signaling pathway. BMC Cardiovasc Disord. 2023;23(1):55. doi: 10.1186/s12872-022-03012-x 36710339 PMC9885634

[10] Kuivaniemi H, Ryer EJ, Elmore JR, Tromp G. Understanding the pathogenesis of abdominal aortic aneurysms. Expert Rev Cardiovasc Ther. 2015;13(9):975–87. doi: 10.1586/14779072.2015.1074861 26308600 PMC4829576

[11] Skovbo Kristensen JS, Krasniqi L, Obel LM, Kavaliunaite E, Liisberg M, Lindholt JS. Exploring drug re-purposing for treatment of abdominal aortic aneurysms: a systematic review and meta-analysis. Eur J Vasc Endovasc Surg. 2024;67(4):570–82. doi: 10.1016/j.ejvs.2023.11.037 38013062

[12] Dalman RL. Limiting AAA With Metformin (LIMIT) Trial Bethesda (MD). Stanford University. National Library of Medicine (US): ClinicalTrials.gov; 2020 [updated 2025 May 9]. Available from: https://clinicaltrials.gov/study/NCT04500756

[13] Pincemail J, Defraigne JO, Cheramy-Bien JP, Dardenne N, Donneau AF, Albert A, et al. On the potential increase of the oxidative stress status in patients with abdominal aortic aneurysm. Redox Rep. 2012;17(4):139–44. doi: 10.1179/1351000212Y.0000000012 22732574 PMC6837517

[14] Obel LM, Diederichsen AC, Steffensen FH, Frost L, Lambrechtsen J, Busk M, et al. Population-based risk factors for ascending, arch, descending, and abdominal aortic dilations for 60-74-year-old individuals. J Am Coll Cardiol. 2021;78(3):201–11. doi: 10.1016/j.jacc.2021.04.094 34266574

[15] Borrell LN. The effects of smoking and physical inactivity on advancing mortality in U.S. adults. Ann Epidemiol. 2014;24(6):484–7. doi: 10.1016/j.annepidem.2014.02.016 24842763

[16] Maggioni AP, Maseri A, Fresco C, Franzosi MG, Mauri F, Santoro E, et al. Age-related increase in mortality among patients with first myocardial infarctions treated with thrombolysis. The Investigators of the Gruppo Italiano per lo Studio della Sopravvivenza nell’Infarto Miocardico (GISSI-2). N Engl J Med. 1993;329(20):1442–8. doi: 10.1056/NEJM199311113292002 8413454

[17] Mathiesen EB, Njølstad I, Joakimsen O. Risk factors for cerebral stroke. Tidsskr Nor Laegeforen. 2007;127(6):748–50. 17363988

[18] Gellert C, Schöttker B, Müller H, Holleczek B, Brenner H. Impact of smoking and quitting on cardiovascular outcomes and risk advancement periods among older adults. Eur J Epidemiol. 2013;28(8):649–58. doi: 10.1007/s10654-013-9776-0 23397516

[19] Maloberti A, Mengozzi A, Russo E, Cicero AFG, Angeli F, Agabiti Rosei E, et al. The Results of the URRAH (Uric Acid Right for Heart Health) project: a focus on hyperuricemia in relation to cardiovascular and kidney disease and its role in metabolic dysregulation. High Blood Press Cardiovasc Prev. 2023;30(5):411–25. doi: 10.1007/s40292-023-00602-4 37792253 PMC10600296

[20] Zhao L, Cao L, Zhao T-Y, Yang X, Zhu X-X, Zou H-J, et al. Cardiovascular events in hyperuricemia population and a cardiovascular benefit-risk assessment of urate-lowering therapies: a systematic review and meta-analysis. Chin Med J (Engl). 2020;133(8):982–93. doi: 10.1097/CM9.0000000000000682 32106120 PMC7176444

[21] Jamil Y, Alameddine D, Iskandarani ME, Agrawal A, Arockiam AD, Haroun E, et al. Cardiovascular outcomes of uric acid lowering medications: a meta-analysis. Curr Cardiol Rep. 2024;26(12):1427–37. doi: 10.1007/s11886-024-02138-y 39352584

[22] Bredemeier M, Lopes LM, Eisenreich MA, Hickmann S, Bongiorno GK, d’Avila R, et al. Xanthine oxidase inhibitors for prevention of cardiovascular events: a systematic review and meta-analysis of randomized controlled trials. BMC Cardiovasc Disord. 2018;18(1):24. doi: 10.1186/s12872-018-0757-9 29415653 PMC5804046

[23] von Elm E, Altman DG, Egger M, Pocock SJ, Gøtzsche PC, Vandenbroucke JP, et al. The Strengthening the Reporting of Observational Studies in Epidemiology (STROBE) statement: guidelines for reporting observational studies. Lancet. 2007;370(9596):1453–7. doi: 10.1016/S0140-6736(07)61602-X 18064739

[24] Lindholt JS, Søgaard R. Population screening and intervention for vascular disease in Danish men (VIVA): a randomised controlled trial. Lancet. 2017;390(10109):2256–65. doi: 10.1016/S0140-6736(17)32250-X 28859943

[25] Lindholt JS, Søgaard R, Rasmussen LM, Mejldal A, Lambrechtsen J, Steffensen FH, et al. Five-Year Outcomes of the Danish Cardiovascular Screening (DANCAVAS) trial. N Engl J Med. 2022;387(15):1385–94. doi: 10.1056/NEJMoa2208681 36027560

[26] Kildemoes HW, Sørensen HT, Hallas J. The Danish national prescription registry. Scand J Public Health. 2011;39(7 Suppl):38–41. 21775349 10.1177/1403494810394717

[27] Lynge E, Sandegaard JL, Rebolj M. The Danish national patient register. Scand J Public Health. 2011;39(7 Suppl):30–3. 21775347 10.1177/1403494811401482

[28] Helweg-Larsen K. The Danish register of causes of death. Scand J Public Health. 2011;39(7 Suppl):26–9. doi: 10.1177/1403494811399958 21775346

[29] Jensen VM, Rasmussen AW. Danish education registers. Scand J Public Health. 2011;39(7 Suppl):91–4. 21775362 10.1177/1403494810394715

[30] Obel LM, Lindholt JS, Leetmaa TH, Dahl JS, Møller JE, Steffensen FH, et al. Individual, expected diameters of the ascending aorta and prevalence of dilations in a study-population aged 60-74 years: a DANCAVAS substudy. Int J Cardiovasc Imaging. 2021;37(3):971–80. 33106964 10.1007/s10554-020-02081-3

[31] Bosco E, Hsueh L, McConeghy KW, Gravenstein S, Saade E. Major adverse cardiovascular event definitions used in observational analysis of administrative databases: a systematic review. BMC Med Res Methodol. 2021;21(1):241. doi: 10.1186/s12874-021-01440-5 34742250 PMC8571870

[32] Ylijoki-Sørensen S, Sajantila A, Lalu K, Bøggild H, Boldsen JL, Boel LWT. Coding ill-defined and unknown cause of death is 13 times more frequent in Denmark than in Finland. Forensic Sci Int. 2014;244:289–94. doi: 10.1016/j.forsciint.2014.09.016 25300069

[33] Stacey BS, Cho JS, Lanéelle D, Bashir M, Williams IM, Lewis MH, et al. A prospective longitudinal study of risk factors for abdominal aortic aneurysm. Physiol Rep. 2024;12(13):e16130. doi: 10.14814/phy2.16130 38946069 PMC11214915

[34] StataCorp. Stata statistical software: release 18. College Station, TX: StataCorp LLC; 2023.

[35] Dupont WD, Plummer WD Jr. Power and sample size calculations. A review and computer program. Control Clin Trials. 1990;11(2):116–28. doi: 10.1016/0197-2456(90)90005-m 2161310

[36] Mosorin M, Juvonen J, Biancari F, Satta J, Surcel HM, Leinonen M, et al. Use of doxycycline to decrease the growth rate of abdominal aortic aneurysms: a randomized, double-blind, placebo-controlled pilot study. J Vasc Surg. 2001;34(4):606–10. doi: 10.1067/mva.2001.117891 11668312

[37] Eilenberg W, Klopf J, Sotir A, Scheuba A, Domenig C, Loewe C, et al. Editor’s choice – metformin to inhibit progression of abdominal aortic aneurysm: a randomised, placebo controlled clinical trial. Eur J Vasc Endovasc Surg. 2025;70(4):417–25. doi: 10.1016/j.ejvs.2025.04.052 40311839

[38] Denmark S. Data for research Copenhagen: statistics denmark; 2024. Available from: https://www.dst.dk/en/kontakt

[39] Yang L, Wu H, Luo C, Zhao Y, Dai R, Li Z, et al. Urate-lowering therapy inhibits thoracic aortic aneurysm and dissection formation in mice. Arterioscler Thromb Vasc Biol. 2023;43(6):e172–89. doi: 10.1161/ATVBAHA.122.318788 37128913

[40] Guzik B, Sagan A, Ludew D, Mrowiecki W, Chwała M, Bujak-Gizycka B, et al. Mechanisms of oxidative stress in human aortic aneurysms–association with clinical risk factors for atherosclerosis and disease severity. Int J Cardiol. 2013;168(3):2389–96. doi: 10.1016/j.ijcard.2013.01.278 23506637 PMC3819986

[41] Obel LM, Diederichsen AC, Steffensen FH, Frost L, Lambrechtsen J, Busk M, et al. High proportions of coexisting aortic dilations call for total aortic scan. J Am Coll Cardiol. 2018;71(7):811–2. 29447746 10.1016/j.jacc.2017.12.013

[42] Dewangga R, Winston K, Ilhami LG, Indriani S, Siddiq T, Adiarto S. Association of metformin use with abdominal aortic aneurysm: A systematic review and meta-analysis. Asian Cardiovasc Thorac Ann. 2024;32(2–3):148–56. doi: 10.1177/02184923231225794 38239055

[43] Kristensen JSS, Dahl M, Larsen KL, Diederichsen ACP, Lindholt JS. Unexplained finding: more than halved aneurysmal growth rates in DANCAVAS trial compared to the VIVA Trial. Aorta (Stamford). 2023;11(05):1–18. doi: 10.1055/s-0044-178794136848907

[44] Skovbo JS, Obel LM, Diederichsen ACP, Steffensen FH, Frost L, Lambrechtsen J, et al. Association of statin treatment and dose with the clinical course of small abdominal aortic aneurysms in men: a 5-year prospective cohort study from 2 population-based screening trials. Circulation. 2025;152(6):384–96. doi: 10.1161/CIRCULATIONAHA.125.074544 40631665

[45] Liisberg M, Diederichsen AC, Lindholt JS. Abdominal ultrasound-scanning versus non-contrast computed tomography as screening method for abdominal aortic aneurysm - a validation study from the randomized DANCAVAS study. BMC Med Imaging. 2017;17(1):14. doi: 10.1186/s12880-017-0186-8 28193267 PMC5307833

[46] Lund KH, Fuglsang CH, Schmidt SAJ, Schmidt M. Cardiovascular data quality in the Danish national patient registry (1977-2024): a systematic review. Clin Epidemiol. 2024;16:865–900. 39678522 10.2147/CLEP.S471335 PMC11645903

[47] Johannesdottir SA, Horváth-Puhó E, Ehrenstein V, Schmidt M, Pedersen L, Sørensen HT. Existing data sources for clinical epidemiology: the Danish National Database of Reimbursed Prescriptions. Clin Epidemiol. 2012;4:303–13. doi: 10.2147/CLEP.S37587 23204870 PMC3508607

[48] Kristensen JSS, Obel LM, Dahl M, Høgh A, Lindholt JS. Gender-specific Predicted normal aortic size and its consequences of the population-based prevalence of abdominal aortic aneurysms. Ann Vasc Surg. 2023;91:127–34. 36563844 10.1016/j.avsg.2022.11.025

[49] Gaffo AL. Gout: clinical manifestations and diagnosis. Available from: https://www.uptodate.com/contents/gout-clinical-manifestations-and-diagnosis?search=gout%20risk%20factors&source=search_result&selectedTitle=1~150&usage_type=default&display_rank=1#H87540416. 2025. Accessed 2025 May 28.

[50] RL D. Overview of abdominal aortic aneurysm. Available from: https://www.uptodate.com/contents/overview-of-abdominal-aortic-aneurysm?search=aaa%20risk%20factors&source=search_result&selectedTitle=2~150&usage_type=default&display_rank=2#H91213688. 2025. Accessed 2025 May 28.

