# Supplementary material for: Association of urate-lowering therapies with abdominal aortic aneurysm growth and clinical events in men: A population-based cohort study
Source: PLoS One. 2026 Jul 31;21(7):e0341242. doi: 10.1371/journal.pone.0341242 (PMC13427005; doi:10.1371/journal.pone.0341242)
Supplement: S1 File — Table of definitions used, origin of data, and specific codes, measurements or timeframes applied in the study. ATC = Anatomical Therapeutic Chemical, ICD10 = International Statistical Classification of Diseases and Related Health Problems 10th Revision, SKS = The Danish Medical Classification System, DANCAVAS = The Danish Cardiovascular Screening trial, VIVA = The Viborg Vascular trial. (PDF) [file pone.0341242.s001.pdf]

Supplementary

Supplementary Table S1 – Definitions

Table of definitions used, origin of data, and specific codes, measurements or timeframes applied in the study. ATC = Anatomical Therapeutic Chemical, ICD10 = International Statistical Classification of Diseases and Related Health Problems 10th Revision, SKS = The Danish Medical Classification System, DANCAVAS = The Danish Cardiovascular Screening trial, VIVA = The Viborg Vascular trial.

Variable	Source	Definition
Abdominal aortic aneurysm	DANCAVAS<br>Or<br>VIVA	Computed tomography scan or ultrasound measurement of the abdominal aorta anterior posterior diameter of:<br>>= 30 and < 55 mm
Ruptured abdominal aortic aneurysm	The Danish National Patient Register	ICD-10:<br>DI713, DI718
Surgery of abdominal aortic aneurysm	The Danish National Patient Register	SKS-codes:<br>KPDG10, KPDG20, KPDG21, KPDG22, KPDG23, KPDG24, KPDG99, KPDQ10, KPDQ20, KPDQ21, KPCQ10, KPCG10.
Diabetes	The Danish National Patient Register<br>and<br>The Danish National Prescription Registry	ICD10:<br>DE10*, DE11*, DE13*<br>or<br>ATC-codes:<br>A10*
Hypertension	The Danish National Patient Register<br>and<br>The Danish National Prescription Registry	ICD10:<br>DI109* DI11*. DI12*, DI13*, DI15*<br>or<br>ATC-codes:<br>C03A*, C03EA*, C08*, C09*.
Chronic obstructive lung disease	The Danish National Patient Register<br>and<br>The Danish National Prescription Registry	ICD10:<br>DJ43*, DJ44*<br>or<br>ATC-codes:<br>R03A*, R03B*
Acute myocardial infarction	The Danish National Patient Register	ICD10:<br>DI21*, DI23*, DI249.

Stroke	The Danish National Patient Register	ICD10:<br>DI61*, DI62*, DI63*, DI649
All-cause mortality	The Danish Cause of Death Registry	ICD10:<br>All
Major Adverse Cardiovascular Event	The Danish National Patient Register	Using modified 3-point MACE criteria: The above definitions for:<br>Myocardial infarction, Stroke or all-cause mortality.
Chronic Kidney Disease	The Danish National Patient Register	ICD10:<br>DI120, DI131, DI132, DN04*, DN06*, DN07*, DN08*, DN14*, DN16*, DN18*.
Dyslipidemia	The Danish National Patient Register	ICD-10:<br>DE78*<br>Or<br>ATC-codes:<br>C10*
Peripheral artery disease	The Danish National Patient Register	ICD-10:<br>DI702*, DI739A, DI739C.
Other large vessels aneurisms	The Danish National Patient Register	ICD-10:<br>DI720, DI722, DI723, DI724, DI712.
Alcohol induced liver disease and alcohol addiction	The Danish National Patient Register	ICD-10:<br>DK70*<br>Or<br>ATC-codes:<br>N07BB*
Lung cancer within 5-years	The Danish National Patient Register	ICD10:<br>DC34*<br>&<br>Within five years of index date
Heart failure	The Danish National Patient Register	ICD10:<br>DI50*, DI110, DI130, DI132
Level of education	The Danish Education Register	International Standard Classification of Education (ISCED).
Anticoagulants	The Danish Prescription Registry	ATC:<br>B01AF*, B01AE*, B01AA*.

Platelet inhibitors	The Danish Prescription Registry	ATC:<br>B01AC*.
NSAID	The Danish Prescription Registry	ATC:<br>M01A*.
Beta Blockers	The Danish Prescription Registry	ATC:<br>C07*.
Thiazides	The Danish Prescription Registry	ATC:<br>C03A*, C03EA*.
Loop-diuretics	The Danish Prescription Registry	ATC-codes:<br>C03C*, C03EB*.
Urate-increasing drugs	The Danish Prescription Registry	The above definitions for:<br>Beta blockers, Thiazides and Loop-diuretics
Angiotensin receptor blockers	The Danish Prescription Registry	ATC:<br>C09C*, C09D*.
Calcium channel blockers	The Danish Prescription Registry	ATC:<br>C08*, C09BB*, C07FB*.
Urate-lowering drugs	The Danish Prescription Registry	The above definitions for:<br>Angiotensin receptor blockers and calcium channel blockers.
Antidiabetics	The Danish Prescription Registry	ATC:<br>A10*.
Insulin	The Danish Prescription Registry	ATC:<br>A10A*.
Metformin	The Danish Prescription Registry	ATC:<br>A10BA02, A10BD02, A10BD03, A10BD07, A10BD08, A10BD10, A10BD11, A10BD13, A10BD14, A10BD15, A10BD16, A10BD17, A10BD18, A10BD20, A10BD22, A10BD23, A10BD25, A10BD26, A10BD27, A10BD28.
Allopurinol	The Danish Prescription Registry	ATC-codes:<br>M04AA01.
Febuxostat	The Danish Prescription Registry	ATC-codes:<br>MO4AA03

Urate-lowering therapies	The Danish Prescription Registry	The above definitions for Allopurinol and Febuxostat.
Statin	The Danish Prescription Registry	ATC-codes:<br>C10AA*.
