# Supplementary material for: Association of urate-lowering therapies with abdominal aortic aneurysm growth and clinical events in men: A population-based cohort study
Source: PLoS One. 2026 Jul 31;21(7):e0341242. doi: 10.1371/journal.pone.0341242 (PMC13427005; doi:10.1371/journal.pone.0341242)
Supplement: S2 File — (PDF) [file pone.0341242.s002.pdf]

Regional Hospital Viborg
Department of Vascular Surgery, Research Section
Helbergs Allé 3
8800 Viborg

For the attention of: Jes Sanddal Lindholdt

Regional Administration Building
Viborg
Health Secretariat
The Scientific Ethical Committee
For the Central Jutland Region
Skrottenborg 26
P.O. Box 21
DK-8800 Viborg
Tel. +45 8728 5000
komite@rm.dk
www.regionmidtjylland.dk

Date: 28.03.2008

Case officer:
MB
Tel. +45 8728 4410
komite@rm.dk
www.komite.rm.dk

Case no.: M-20080028

Regarding the project "Preventive circulatory examination of men aged 65–74 years in the Central Jutland Region."

The Regional Committee on Health Research Ethics for the Central Jutland Region finds that the above-mentioned project is in accordance with Act no. 402 of 28 May 2003 on a system of research ethics committees and the processing of biomedical research projects.
The project is hereby permitted to be initiated.

The Committee has based its permission on project material received on 25 January 2008 and 14 February 2008, as well as revised project material received on 14 March 2008.

The Committee has noted that the project is expected to be completed on 1 May 2019.

If significant changes are made to the trial protocol during the conduct of the project, these must be reported to the Committee. The changes may only be implemented after approval by the Committee.

The Committee must be notified immediately if serious adverse reactions or serious events occur during the project. Once annually throughout the entire trial period, the Committee must be sent a list of all serious adverse reactions and serious events that have occurred during the project period, together with a report on the safety of the trial participants.

No later than 90 days after the completion of the project, the Committee must be notified that the project has been completed. The final research report or publication must be submitted to the Committee as soon as possible thereafter. In addition, the Committee may carry out more detailed follow-up of selected projects.

If the project is terminated earlier than planned, this, as well as the reason for such termination, must be reported to the Committee within 15 days.

All inquiries regarding the project should be addressed to the Committee's secretariat, stating the Committee's journal number.

Yours sincerely

Marie Bartholdy
Executive Officer
