# Supplementary material for: Association of urate-lowering therapies with abdominal aortic aneurysm growth and clinical events in men: A population-based cohort study
Source: PLoS One. 2026 Jul 31;21(7):e0341242. doi: 10.1371/journal.pone.0341242 (PMC13427005; doi:10.1371/journal.pone.0341242)
Supplement: S3 File — (PDF) [file pone.0341242.s003.pdf]

Senior Consultant, Research
Lecturer, ph.d. Axel Diederichsen
Odense Universitetshospital
Department of Cardiology
Sdr Boulevard 29
5000 Odense C

**De Videnskabsetiske Komiteer
for Region Syddanmark**

komite@rsyd.dk

30. april 2014

Projekt-ID: S-20140028
CKH/bss

<u>Research project</u>: <u>The Danish Cardiovascular Screening (DANCAVAS} Trial - A combined, randomized clinically controlled multicentre intervention trial and cohort study</u>

Final approval

The Regional Committee on Health Research Ethics for the Region of Southern Denmark hereby confirms receipt of the email dated 14 April 2014, submitted in response to the Committee's decision of 2 April 2014, in which conditions for approval of the project were stipulated.

Decision

The conditions for approval are considered to be fulfilled. The project is therefore finally approved. The decision has been made pursuant to Act No. 593 of 14 June 2011 on the ethical review of health research projects.

The approval applies to the notified trial sites, the notified principal investigators in Denmark, and the stated trial period. The Committee assumes that the principal investigator will ensure that the other participants in the project are informed of the Committee's decision in this matter.

The approval is valid until **30** April 2025, and the following documents have formed the basis for the assessment:

- Signed application, dated 17 February 2014
- Protocol received on 15 April 2014
- Participant information received on 15 April 2014
- Informed consent form received on 19 February 2014
- Response letter dated 14 April 2014

The approval applies to the notified trial sites and the notified principal investigator in Denmark

Initiation of the project in contravention of the approval may be punishable by a fine or imprisonment pursuant to section 41 of the Committee Act.

Remarks
The approval includes permission to disclose information from patient records to the researcher in accordance with section 46(1) of the Danish Health Act. The permission covers disclosure of the information listed in the protocol.

Amendments
If significant amendments are made to the protocol material during the conduct of the project, these must be reported to the Committee in the form of addendum protocols. The amendments may only be implemented after approval by the Committee, cf. section 27(1) of the Committee Act.

Addendum protocols must be submitted electronically via www.drvk.dk using the already assigned notification number and access code.

Significant amendments include, inter alia, changes that may affect participant safety, interpretation of the scientific documentation on which the project is based, as well as the conduct or management of the project. This may include changes to inclusion and exclusion criteria, study design, number of participants, trial procedures, treatment duration, outcome measures, changes to principal investigators or trial sites, as well as substantive changes to the written information material provided to participants.

Where new information leads the researcher to consider changing procedures or terminating the trial, the Committee must be informed accordingly.

Adverse reactions and events
Ongoing reporting
The Committee must be notified immediately if suspected serious, unexpected adverse reactions or serious adverse events occur during the project, cf. section 30(1) of the Committee Act. The notification must be accompanied by comments on any potential consequences for the trial. Only adverse reactions and events occurring in Denmark must be reported.
Notification must be made no later than 7 days after the sponsor or principal investigator becomes aware of the case.

For reporting purposes, a form available at www.dnvk.dk may be used. The form with appendices may be submitted electronically using a digital signature or on CD-ROM.

Annual reporting
Once annually throughout the entire trial period, the Committee must receive a list of all suspected serious (expected and unexpected) adverse reactions and serious adverse events that have occurred during the trial period, together with a report on participant safety, cf. section 30(2) of the Committee Act.

The material must be in Danish or English.

For reporting, a form available at www.dnvk.dk must be used. The form with appendices may be submitted electronically using a digital signature or on CD-ROM.

Completion
The principal investigator must notify the Committee no later than 90 days after completion of the project, cf. section 31(1) of the Committee Act. The project is considered completed when the last participant has completed the trial.

If the project is terminated earlier than planned, a justification must be submitted to the Committee no later than 15 days after the decision is made, cf. section 31(2) of the Committee Act.

If the project is not initiated, this and the reason must be communicated to the Committee. The Committee requests a copy of the final research report or publication, cf. section 28(2) of the Committee Act. In this connection, attention is drawn to the obligation to publish negative, positive, and inconclusive trial results, cf. section 20(1)(8) of the Committee Act.

Please state the Project ID when submitting material related to the project. Inquiries concerning the project may be addressed to the Committee Secretariat.

Supervision
The Committee supervises that the project is conducted in accordance with the approval, cf. sections 28 and 29 of the Committee Act.

The following Committee members participated in the meeting review on 18 March 2014:

- Birger Møller
- Niels Illum
- Jeppe Gram
- Rikke Kroger Jensen
- Hanne Vestergaard
- Susanne Eilersen
- Niels Erik Søndergaard
- Herdis Hanghøi
- Sonny Berthold
- Pia Tørving
- Peter Christensen

The case has been reviewed and finally approved by the Chair of the Committee, Consultant Cardiologist, Dr. Med. Birger Møller, on 29 April 2014.

On behalf of the Committee
Kind Regards

Birger Møller / Claus Kvist Hansen
Formand Sekretariatsleder

Copy to: In accordance with an agreement between the National Committee on Health Research Ethics and the regional committees, a copy of the decision is also forwarded to:
The Danish Institute for Radiation Protection, Knapholm 7, 2730 Herlev

Consultant Cardiologist, Professor, Dr. Med., PhD
Jes S. Lindholt, Department of Thoracic Surgery T, OUH
