# Supplementary material for: Association of urate-lowering therapies with abdominal aortic aneurysm growth and clinical events in men: A population-based cohort study
Source: PLoS One. 2026 Jul 31;21(7):e0341242. doi: 10.1371/journal.pone.0341242 (PMC13427005; doi:10.1371/journal.pone.0341242)
Supplement: S4 File — (PDF) [file pone.0341242.s004.pdf]

**De Videnskabsetiske Komitéer**
**for Region Syddanmark**

komite@rsyd.dk

Senior Consultant, Research Lecturer, PhD
Axel Diederichsen
Odense University hospital
Hjertemedicinsk Afdeling B Sdr Boulevard 29
5000 Odense C

7. februar 2016

Projekt-ID: S-20140028
SRJ

**Regarding research project: The Danish Cardiovascular Screening (DANCAVAS) Trial**
**- A combined, randomized clinically controlled multicentre intervention trial and cohort study**

The Regional Committee on Health Research Ethics for the Region of Southern Denmark received, on 29 January 2016, a notification of an addendum with notification number 52074.

Addendum no. 5 concerns:
The DANCAVAS study is to be expanded to include a group of men aged 60–64 years.

The Committee has no comments on the submitted material and is able to approve the above-mentioned addendum.

The approval covers the following documents:

- Participant information – men aged 60–64 years
- Addendum protocol

The case has been reviewed and approved by the Chair of Committee 1, Dr. Med. Birger Møller, on 6 February 2016.

Kind regards

Signe Riktrup Jensen

Signe Riktrup Jensen
Chief consultant

Copy for: Senior Consultant, professor, dr. med., ph.d., Jes Sanddal Lindholt, OUH, Thoraxkirurgisk Afdeling T, Sdr. Boulevard, 5000 Odense C Jes.sanddal.lindholt@rsyd.dk
