# Supplementary material for: Association of urate-lowering therapies with abdominal aortic aneurysm growth and clinical events in men: A population-based cohort study
Source: PLoS One. 2026 Jul 31;21(7):e0341242. doi: 10.1371/journal.pone.0341242 (PMC13427005; doi:10.1371/journal.pone.0341242)
Supplement: S5 File — (PDF) [file pone.0341242.s005.pdf]

Machine Translated by Google

Preventive cardiovascular screening of men aged 65–74 years in the Central Denmark Region

PhD student[1] Senior Consultant in Research Søren Paaske Johnsen[2], Chief Consultant Eskild W. Henneberg[1], Professor Henrik Toft Sørensen[2], Senior Consultant in Research Jes S. Lindholt (Principal Investigator)[1]

[1] Research Section, Department of Vascular Surgery, Viborg Regional Hospital
[2] Department of Clinical Epidemiology, Aarhus University

Lay Summary

Preventive screening for abdominal aortic aneurysm (AAA) in men aged 65–74 years using ultrasound scanning has proven to be a simple and safe method to prevent rupture.
Approximately 4% of men have such an asymptomatic dilation. The vast majority of aneurysms (80–85%) diagnosed through screening are too small to pose an immediate risk; however, up to half of these may grow to a dangerously large size requiring surgical intervention to prevent rupture. Preventive surgery carries a mortality risk of approximately 3%, compared with around 90% in the event of rupture.

There is increasing evidence that aneurysm growth can be slowed through smoking cessation, low-dose aspirin, cholesterol-lowering medication, and antihypertensive treatment—preventive measures that simultaneously reduce the increased cardiovascular risk faced by these patients.
Furthermore, such preventive screening can likely be combined advantageously with other preventive examinations.
Approximately 5–10% of men over the age of 60 show signs of early peripheral arterial disease (PAD) in the lower limbs. This condition is often benign with respect to limb outcomes—although a proportion ultimately require amputation—but more importantly, it is a strong risk marker for thrombotic events elsewhere in the body. Approximately 25–30% of these individuals will die from cardiovascular disease within five years, and an even larger proportion will be hospitalized due to cardiovascular disease. Cholesterol-lowering interventions, smoking cessation, low-dose acetylsalicylic acid, increased physical activity, healthy dietary habits, and blood pressure control reduce this excess risk of cardiovascular disease and mortality by at least 20–30%.
Early PAD can be diagnosed reliably and without adverse effects by measuring ankle blood pressure and comparing it with arm blood pressure. This can be done quickly, reliably, and non-invasively using a portable Doppler ultrasound device and blood pressure cuff, making it suitable for screening purposes.
There are thus well-documented public health benefits to be gained from screening for and preventing vascular diseases—both in terms of avoided premature deaths and reduced hospital admissions and amputations. There will also be indirect positive effects for hospitals through reduced pressure on surgical and intensive care capacity in vascular surgery departments, cardiology departments, and neurological departments.
It therefore appears highly relevant to offer combined screening for abdominal aortic aneurysm, peripheral arterial disease, and hypertension, although the benefits and costs of such a program have not yet been fully evaluated.
The primary objective of the study is therefore to assess the benefit and cost-effectiveness of a combined cardiovascular screening program for men aged 65–74 years through a randomized,

Machine Translated by Google

clinically controlled trial (lottery-based trial), in which half of 40,000 men aged 65–74 years are invited to preventive screening at regional hospitals, while the other half serve as a control group.
The principal investigator will train six nurses in screening methods, information requirements, and blood sampling. These nurses will bring mobile equipment to regional hospitals, where examinations will be conducted along with follow-up for positive findings.
The project secretary will continuously invite men for screening at their “regional” hospital with 2–4 weeks' notice. A brief lifestyle and health questionnaire will be enclosed, along with the option to decline participation in writing or request an alternative appointment. Participants will receive initial oral information, including the option of a reflection period of a few weeks; if this is waived, written informed consent will be obtained before the examinations are conducted.
Men with positive findings will be offered a follow-up informational consultation within one week, during which findings are explained and the aforementioned preventive measures initiated. In addition to lifestyle counseling, it will often be necessary to initiate treatment with low-dose aspirin and cholesterol-lowering medication. This medication carries a small risk of gastric irritation or bleeding, as well as muscle pain. These adverse effects occur in only a few per thousand individuals and must be weighed against the expected benefit. During this consultation, consent will also be obtained for blood sampling for a biobank for prognostic studies, conducted in connection with cholesterol testing. Finally, annual follow-up will be offered.

The participants are mature men capable of making fully independent decisions regarding participation, and emphasis is placed on providing as comprehensive an explanation as possible in the invitation, which is sent 2–4 weeks prior to the scheduled examination. After oral information, time for consideration will be provided. In the event of a positive finding, an in-depth outpatient consultation will be offered within one week. Thus, opportunities for reflection are provided at multiple stages, and participants are explicitly encouraged to bring a companion if they wish.

Offering screening for AAA is known to reduce quality of life in the period prior to examination, but this normalizes immediately afterwards—except in cases of positive findings. It is unclear whether this is due to the diagnosis itself or comorbidity, but the reduction is modest. This is likely also the case for PAD. This "side effect" must be weighed against the substantial preventive benefit achieved.

More problematic in AAA screening is the risk of death due to rupture in conservatively treated cases, as well as perioperative deaths among operated patients whose aneurysms might never have ruptured. There is an immediate operative mortality risk of approximately 3%, which must be weighed against a rupture-related mortality risk of around 90%. This ethical dilemma is serious but currently unavoidable. Exploration of the planned biobank aims, among other objectives, to develop prognostic models. Participation in the biobank exposes participants to a very small risk of infection or phlebitis associated with blood sampling, which is in any case necessary for cholesterol determination.

Effectiveness and cost analyzes will be conducted using registry-based methods and will therefore not impose any burden on participants.
The project is supported by the EU's Seventh Framework Programme. No remuneration is provided to participants.

The biobank will be maintained for 10 years, after which the samples will be anonymised.

Machine Translated by Google

Background

Screening for abdominal aortic aneurysm (AAA) in men aged 65–74 years has been shown to reduce AAA-related mortality. Research from Regionshospitalet Viborg, published in the *British Medical Journal* in March 2005, demonstrated that it is sufficient to screen 352 Danish men over the age of 65 for AAA to prevent one death due to AAA rupture within the subsequent five years (1). The benefit has also been shown to be reasonably proportionate to the costs in randomized studies (2–4). However, there are currently no recommendations for AAA screening in Scandinavia, although a Danish health technology assessment may be forthcoming.

The vast majority of AAAs diagnosed through screening are too small to pose an immediate risk, but up to half of them expand to sizes requiring surgical intervention. Increasing evidence suggests that aneurysm growth can be slowed through smoking cessation (5;6), low-dose aspirin (7;8), statin therapy (9), and ACE inhibitor treatment (10)—preventive measures that simultaneously reduce the increased cardiovascular risk faced by these patients (11). The aforementioned screening studies did not implement such preventive measures; consequently, the benefit and thus the cost-effectiveness may potentially be even greater.

In addition, invitations to AAA screening could advantageously be combined with further preventive examinations. International scientific studies indicate that approximately 5–10% of men over the age of 60 show signs of peripheral arterial disease (PAD) in the lower extremities, with prevalence increasing with age (12–14). Of these individuals, approximately 25–30% will die from cardiovascular disease within a five-year period, and an even larger proportion will be hospitalized due to cardiovascular disease (14–16). Cholesterol-lowering interventions, smoking cessation, low-dose acetylsalicylic acid, increased physical activity, healthy dietary habits, and blood pressure control reduce this excess risk of cardiovascular disease and death by at least 20–25% (14;17–19).

PAD can be diagnosed quickly, reliably, and non-invasively using a portable Doppler ultrasound device and a blood pressure cuff. PAD is defined as an ankle–brachial systolic blood pressure index (ABI) of <90% (14).

There are therefore well-documented health benefits to be gained from screening for and preventing vascular diseases, both in terms of avoided premature deaths and avoided hospital admissions and amputations. There will also be a secondary positive effect for hospitals through reduced pressure on surgical capacity and intensive care capacity in vascular surgical departments.
It therefore appears highly relevant to offer combined screening for AAA, PAD, and hypertension, although the benefits and costs of such a program have not yet been fully elucidated.

Purpose:

1. The primary objective of the study is to assess the effectiveness and cost-effectiveness of a combined cardiovascular screening program for men aged 65–74 through a randomized, clinically controlled trial.

2.Secondary objectives include:
a. A prevalence study of abdominal aortic aneurysm (AAA), peripheral arterial disease (PAD), and previously undiagnosed hypertension among men aged 65–74. This will be a cross-sectional study conducted in connection with the screening offer, as well as a comparison with the corresponding AAA prevalence identified in the screening study conducted in Viborg County during 1994–1998,

Machine Translated by Google

as both the frequency of planned operations and emergency operations for AAA has increased over the past decade, possibly indicating a rising prevalence.

b. An evaluation of the effect of smoking cessation campaigns and treatment with aspirin and statins on AAA growth rate, need for surgery, and cardiovascular mortality. This will be achieved by comparison with the AAA cohort diagnosed through screening in the period 1994–1998, which was not offered any form of preventive intervention.

c. A contemporary prognostic study of patients with PAD who are offered relevant preventive measures.

d. Predictor studies. Previous studies have identified several moderate serological predictors of the clinical course of non-surgically treated AAAs. These will be combined in regression models and validated. For this purpose, a biobank will be established with blood samples collected at the time of diagnosis and at the first follow-up. A corresponding biobank will be established for PAD.

e. Assessment of the psychological consequences of cardiovascular screening, evaluated using the SF-12 questionnaire (20).

Target population (men aged 65–74 years)

In order to maximize the potential to demonstrate an effect, men aged 65–74 years are screened due to the high prevalence of disease and a long expected remaining life expectancy. Screening men older than 74 years would likely yield a high prevalence of AAA and PAD; however, mortality from other diseases is high and the acceptance rate is low. Screening men younger than 65 years would result in lower mortality, but also lower prevalence and a lower rupture rate.

The prevalence of PAD and AAA is lower in women and occurs later in life; therefore, offering screening to women is expected to result in lower participation and higher mortality from other causes, and consequently a lower overall effect.

As of 1 January 2008, there will be approximately 40,000 men aged 65–74 years in the Central Denmark Region. These men constitute the target population for the study, with no exclusion criteria.

Randomization

Civil registration number (CPR), name, and address information are provided by the Department of Clinical Epidemiology (KEA), which also performs the randomization in blocks of approximately 1,000 individuals in order to avoid long delays between data extraction and invitation.

Half of the population is invited to undergo cardiovascular screening for PAD, AAA, and hypertension, while the other half serves as the control group, as there are currently no Scandinavian recommendations for screening for AAA. Using Epidata, name and address information are merged with the screening invitation.

Screening

The project leader trains six nurses in ankle–brachial index (ABI) measurement and ultrasound (US) examination of the aorta. These nurses operate in three teams, bringing mobile handheld Doppler devices, blood pressure cuffs, and mobile ultrasound scanners to the regional hospitals.

One team will conduct screening at Herning, Holstebro, Ringkøbing, Lemvig, and Tarm Hospitals.

A second team will screen at Randers, Silkeborg, Kjellerup, Viborg, and Skive Hospitals, and a third team will screen in Aarhus, Skanderborg, and Horsens.

Machine Translated by Google

The project secretary continuously invites participants to their "regional" hospital using names and addresses from the randomisation files. To ensure a stable and even attendance rate, 74-year-old men are invited together with 65-year-old men, 73-year-old men together with 66-year-old men, and so on (see Appendix 1 – Invitation). Invitees to the full cardiovascular screening are asked to wear appropriate footwear. The invitation includes the option to decline the offer in writing or to request an alternative appointment. Non-responding men are re-invited once.
Finally, a short questionnaire on smoking habits, medical history, walking-related pain, and medication use is enclosed and requested to be completed and brought to the screening examination (Appendix 2). At randomisation, 500 participants are selected to complete the SF-12 questionnaire, and a further 500 are selected to complete the WHO-5 questionnaire after the screening examination.
Following initial information and the collection of written informed consent using the Central Ethics Committee's standard consent form for competent adults (Appendix 3), the man is instructed to lie on the examination couch with the abdominal skin exposed. The ultrasound transducer is placed longitudinally just above the umbilicus and slightly to the left. The aorta is visualized. In the presence of a dilatation, the maximum perpendicular anteroposterior (AP) diameter is measured. If no dilatation is present, the AP diameter is measured two centimeters above the bifurcation.
Simultaneously, peripheral pulse conditions are recorded by the second nurse, and automated brachial blood pressure is measured while ankle blood pressure is determined by capturing the Doppler signal from the dorsalis pedis artery (ADP). The blood pressure cuff is inflated until the signal disappears and then slowly deflated until the signal reappears. The mean pressure of these two measurements is recorded, and the same procedure is performed for the posterior tibial artery (ATP). The procedure is subsequently repeated on the contralateral extremity.
In cases of peripheral arterial disease (PAD) (defined as ABI ÿ 0.9) or abdominal aortic aneurysm (AAA) (defined as a maximal infrarenal aortic diameter ÿ 30 mm), the nurse informs the “patient” of the finding and its likely consequences, but asks him to attend a further consultation. Verbal and written information on data registration and the biobank is provided, including information about the option to bring a companion to the follow-up consultation (Appendix 4). In cases of suspected hypertension without PAD or AAA (defined as blood pressure >140/90 mmHg), the patient is encouraged to have this clarified and, if necessary, treated by his general practitioner, as blood pressure monitoring and treatment are considered best managed in that setting.

Follow-up consultation in case of a positive finding

The most important aspect of this consultation is calm and thorough information about the finding and recommendations regarding its consequences.

PAD: Information is provided regarding the relatively benign prognosis in terms of limb survival and the necessity of initiating effective preventive measures in the form of walking exercise/physical activity, smoking cessation, a low-fat diet, aspirin therapy, and statin treatment.
Cholesterol levels are measured. If cholesterol exceeds 4.0 mmol/L, it is not expected to decrease below 3.5 mmol/L with dietary changes alone; therefore, a prescription for simvastatin 40 mg daily is provided. Annual follow-up including ABI measurement is offered. WHO-5 is completed at these follow-up visits (21).
AAA: The aorta is reassessed, the finding is digitally stored, and the degree of calcification is determined. Information is provided about the natural history of AAA and the need to initiate effective preventive measures in the form of physical activity, smoking cessation, a low-fat diet,

Machine Translated by Google

aspirin therapy, and statin treatment, in accordance with the consensus guidelines of the European Society of Cardiology 2007 (22). For AAA measuring ÿ50 mm in maximum diameter, referral for CT scanning and further vascular surgical evaluation and surveillance or surgery is made. Annual follow-up including ultrasound examination is offered. WHO-5 is completed after the consultation and at subsequent follow-up visits, as well as annually after AAA surgery (23).

For both patient groups, written consent is obtained for participation in the biobank and for data registration using the CVK's pre-printed addendum to the consent form (Appendix 3). Blood samples are collected and centrifuged, labels are applied, samples are refrigerated, and upon return to the primary hospital frozen at ÿ80°C, except for one sample used for cholesterol determination.

If cholesterol exceeds 4.0 mmol/L, it is not expected to decrease below 3.5 mmol/L with dietary modification alone; therefore, a prescription for simvastatin 40 mg daily is subsequently sent by the medical project leader.

Steering committee

A steering committee is established consisting of the project leader and a representative from the Department of Clinical Epidemiology. In addition, a vascular surgeon from each of the two affected vascular surgery departments in the region participates.

Facilities (a room with a table, an examination couch, two chairs, and a power supply) are arranged by the project leader. On-call physician facilities at the region's hospitals are primarily used.

The two vascular surgeons ensure availability of preoperative outpatient appointments and surgical capacity within one month.

Outcome measures and statistical analyses

The primary outcome measures are all-cause mortality, cardiovascular mortality, and AAA-related mortality. Secondary outcome measures include cardiovascular-related hospital services and the associated costs, calculated according to the relevant DRG tariffs.

The entire population, both the control group and the screening group, is followed for 10 years.

Information on deaths, including date of death, is obtained from the Civil Registration System (CPR). Information on outpatient hospital visits and hospital admissions due to cardiovascular conditions, including amputations, is obtained from the National Patient Register. Information on cause of death is obtained from the Cause of Death Register and classified according to whether deaths were AAA-related and/or cardiovascular-related. Vascular surgical procedures are identified in the "Karbase" registry. This extensive follow-up is conducted after 3, 5, and 10 years.

Total mortality, cardiovascular mortality, AAA-related mortality, and first cardiovascular hospital event are compared between the two groups using Cox proportional hazards regression analysis, allowing estimation of risk ratios.

Cost-effectiveness is calculated using discounting and adjustment for quality of life.

Power calculations

With a 75% attendance rate and a PAD prevalence of 8%, assuming a five-year total mortality of 28% compared with 15% in the control group, a relative risk (RR) of 0.93 is calculated. With a 5% significance level and a sample size of 2 × 20,000, the statistical power is estimated at 85%.

With a 75% attendance rate and 8% PAD prevalence, where 70% experience a cardiovascular-related hospital admission within five years compared with 45% in the control group, an RR of 0.93 is calculated. With a 5% significance level and a sample size of 2 × 20,000, the statistical power is estimated at 99%.

Machine Translated by Google

The expected absolute reduction in mortality is based on survival data from the screening study of 65–73-year-old men in Viborg County, as follows: an expected 10-year mortality of 36% in the control group, corresponding to 7,200 deaths. If the relative mortality with a screening victim is 0.93, as calculated, this corresponds to 6,696 deaths. This represents a difference of 504 prevented deaths, corresponding to an absolute mortality risk reduction of 2.5%.
The power calculations are conservative, as they are based solely on the expected benefit of PAD screening. A recent meta-analysis of the four existing randomized trials showed that screening for AAA results in a significant absolute mortality reduction of 3%. However, these studies did not offer general cardiovascular prevention to screening-positive individuals. One third of men with screen-detected AAA also have signs of PAD. A cautious estimate of the potential combined benefit would therefore be an absolute mortality risk reduction of at least 4–5%.

Financial and budget administration
The acquired funds are administered by the vascular surgery unit, where the nurses are employed, ensuring that remuneration is provided in accordance with collective agreements via the payroll and personnel office at Regionshospitalet Viborg. Handheld Doppler devices, blood pressure cuffs, cholesterol meters, ultrasound scanners, and other materials are purchased by the unit. Postal expenses are reimbursed to the hospital's postal account.
The expenses are primarily financed through EU funding under FP7, while PAD screening is sought to be financed by the Central Denmark Region.
Project leader Jes Lindholt initiated the research and has no financial affiliation with any foundations or the EU. No honorarium or travel reimbursement is provided to study participants.

Expected number of examinations, operations, and follow-up visits
Three screening teams will be established—one covering the former Aarhus County, and one each corresponding to the former Viborg and Ringkøbing Counties. At each site, 34 men are invited daily at 10-minute intervals (with an expected primary participation rate of 70%). Non-responders are invited at five-minute intervals. In this way, the target population is expected to be examined within 1.5 years.
Examinations take place four days per week from 9:00 to 12:00 and from 12:30 to 15:00. The nurses' positions will therefore correspond to 30 hours per week, including transport time. From Monday to Thursday, approximately 150 examinations are performed, with an expected finding of approximately 4 AAA cases and 8 PAD cases per site.
Patients attend outpatient consultations on the fifth day of the week in the same room from 9:00. Thirty minutes are allocated per patient. Every tenth AAA case is expected to be referred for surgical evaluation, of which 90% are expected to undergo surgery. This corresponds to 1–2 additional AAA operations per month per department.
The departments' consultant vascular surgeons foresee no operative or nursing-related capacity problems, primarily because emergency operations require several times longer operative and hospitalization times—especially in intensive care units—compared with elective procedures.

Information and Consent
The study is based on the offer of a screening examination. The written information thus constitutes the offer of the examination (Appendix 1). This is sent approximately 14 days before the pre-booked examination appointment with information that further details are available. This combined information and invitation bears the project's title and begins with a request to participate in a biomedical research project.
This is followed by a description of the study's purpose and background, the study method, what it is

Machine Translated by Google

new about the study, and indirectly the potential benefit to the participants themselves, as well as that the study can only be considered discontinued if the offer is introduced generally through political channels. Finally, participants are encouraged to read the attached supplement, "Participant Rights in a Biomedical Research Project."
Upon attendance, the attending man and any accompanying person are informed about the background, method, and possible consequences of a positive finding by the project nurse. This takes place in a calm setting, with the possibility of a reflection period of 1–2 weeks. If this is not desired, written consent is obtained using CVK's pre-printed consent form for competent adults, after which the examinations are performed (Appendix 3). In the event of a PAD or AAA finding, the nurse informs the "patient" about this and the likely consequences, but asks them to attend for further information. The same nurse also provides verbal and written information about data registration and biobank participation, with the option to bring a companion to the detailed consultation (Appendix 4).
The most important aspect of this nurse-led consultation is to calmly inform the participant of the finding and recommend the consequences. Information is provided about the relatively benign prognosis regarding limb survival and prevention of aortic rupture, along with the necessity of implementing effective preventive measures such as walking/exercise, smoking cessation, low-fat diet, aspirin, and possibly statin therapy. Written information is also provided (Appendix 5). Whenever possible, written consent is obtained for biobank participation and data registration using CVK's pre-printed consent addendum (Appendix 6).

Ethical Considerations

The project has been reported to the Regional Scientific Ethics Committee of Central Jutland.
The victim of AAA screening is known to reduce quality of life in the period before the examination but tends to normalize shortly afterwards, except in cases of positive findings. It is unclear whether this is due to the diagnosis itself or comorbidity, but the reduction is minor. This is likely also the case with PAD. This "side effect" must be weighed against the preventive benefit achieved.
A more serious concern is that AAA carries a risk of death due to rupture in conservatively treated cases, as well as perioperative death in operated cases that may never have ruptured. This ethical problem is serious but currently unsolvable.
The planned biobank research aims, among other things, to develop a prognostic model. Participation in the biobank exposes participants to a very small risk of infection and thrombophlebitis related to blood sampling, which is necessary for cholesterol determination. Participants are mature men capable of making an independent decision about whether to accept the offer, and emphasis is placed on providing as comprehensive an explanation as possible in the invitation (see "Invitation to Screening," Appendix 1). In the case of a positive finding, participants are given a request for data registration and biobank participation (Appendix 3), and all with a positive finding are offered a detailed outpatient consultation at the earliest opportunity, along with follow-up examinations. This allows reflection time for participation, and participants are informed that they may bring a companion, preferably a spouse or cohabitant. During the consultation, participants are informed about prognosis and necessary preventive measures.
In addition to advice and guidance, it is often necessary to start treatment with aspirin and cholesterol-lowering medication. This medication carries a minor risk of excess stomach acid or gastrointestinal bleeding, as well as muscle pain, which occurs in very few cases per thousand, and should be compared to the risk reduction achieved.
A particular dilemma arises when an AAA becomes operable, as this carries an immediate surgical mortality risk of 3%, which must be weighed against the ~90% risk of death if rupture occurs.
When there is an indication for AAA surgery, participants are informed of the risks of surgery

Machine Translated by Google

versus conservative treatment, and the operation date is scheduled. The project leader is experienced in this role.
The biobank is maintained for 10 years, after which the samples are anonymised.

Quality Assurance and Training
The project leader trains nurses during the initial phase so that they do not perform examinations independently until acceptable inter-individual variation is achieved: 3–5 mm for aortic scanning and 15% for ABI (ankle-brachial index) measurements. As quality assurance, the examination quality will be validated semi-annually.

Data Registration
Data registration is conducted using computer-based entry and numeric coding, partly for convenience and partly to protect the participant's data, as their CPR number and name are registered. Registration is performed by the project leader. The computer with access to the information is kept in a locked room at the Research Section of Viborg Regional Hospital and requires a password to access. The database is stored on the hospital's internal drive, preventing data loss in case of PC failure. Only the project leader and project secretary have access to these data. The information will not be disclosed to public offices, insurance companies, pension funds, or similar entities.

Consent for data registration is confirmed in writing by the "patient" at the first outpatient consultation. The project is reported to the Danish Data Protection Agency through RM's centralized reporting.

Publications
The project is planned to be published in an international journal with the author order as stated on page 1. Both positive and negative findings will be published.

Five-year budget

Expense Item	Quantity	Unit Price (DKK)	Total (DKK)
Equipment			
Mobile UL scanners	3	250,000	750,000
Handheld Dopplers*	3	5,700	17,100
Automatic BP devices (Omron M6)*		460	2,760
Manual BP devices*	3	250	750
Mobile blood centrifuge (Sigma 2-6)	3	12,750	38,250
Mobile cholesterol meter*	3	2,000	6,000
Staff			
PhD student	3 444,000/year 3 300,000/		1,332,000
Nurses (AAA screening) (18 months)	year 3 300,000/year 1		1,350,000
Nurses (PAD screening) (18 months)*	250,000/year 1 300,000/		1,350,000
Project secretary	year ½ 450,000/year 1½		1,250,000
Follow-up project nurse (3½ years)	20,000/year		1,050,000
Project leader (48 months)			675,000
PhD fee			30,000

Machine Translated by Google

Operational Costs			
Primary invitations 20,000 6.00 Secondary invitations 6,600 6.00			120,000
Cholesterol tests* 2,800 10.00 Travel allowance (6 x 20 km x 350 days)			39,600
42,000 3.38 Travel allowance (2 x 20 km x 70 days) 2,800 3.38 Quality			28,000
checks 3 10,000 Information to own GP 1,400 6.00 Database costs			142,000
			9,500
			30,000
			8,400
			500,000
**Total**			**10,208,860**
**AAA screening**			**8,758,250**
FP7 (FAD)			6,130,775
30% co-financing from RH Viborg			2,627,475
***PAD screening**			**1,404,611**

Regarding the costs for PAD screening: These are incurred over the first 18 months for the initial purchase of equipment and the ongoing operational costs for the additional project nurses. The expense items are marked with * in the table above. Distributed over the years, they are as follows:

**PAD Screening**	2008 (3 months)	2009 (12 months)	2010 (3 months)
Equipment	26,610		
Salary + cholesterol tests	229,667	918,667	229,667
**Total**	**256,277**	**918,667**	**229,667**

Machine Translated by Google

Reference List

(1) Lindholt JS, Juul S, Fasting H, Henneberg EW. Screening for abdominal aortic aneurysms: single center randomized controlled trial. BMJ 2005 Apr 2;330(7494):750.
(2) Lindholt JS, Juul S, Fasting H, Henneberg EW. Cost-effectiveness Analysis of Screening for Abdominal Aortic Aneurysms Based on Five Year Results from a Randomized Hospital Based Mass Screening Trial. Eur J Vasc
Endovasc Surg 2006 Apr 5;32:9-15.
(3) Lindholt JS, Juul S, Fasting H, Henneberg EW. Preliminary ten year results from a randomized single center mass screening trial for abdominal aortic aneurysm. Eur J Vasc Endovasc Surg 2006 Dec;32(6):608-14.
(4) Kim LG, RA PS, Ashton HA, Thompson SG. A sustained mortality benefit from screening for abdominal aortic aneurysm 2. Ann Intern Med 2007 May 15;146(10):699-706.
(5) Smoking, lung function and the prognosis of abdominal aortic aneurysm. The UK Small Aneurysm TrialParticipants. Eur J Vasc Endovasc Surg 2000 Jun;19(6):636-42.
(6) Cornuz J, Sidoti PC, Tevaearai H, Egger M. Risk factors for asymptomatic abdominal aortic aneurysm: systematic review and meta-analysis of population-based screening studies. Eur J Public Health 2004 Dec;14(4):343-9.
(7) Fontaine V, Jacob MP, Houard X, Rossignol P, Plissonnier D, ngles-Cano E et al. Involvement of the mural thrombus as a site of protease release and activation in human aortic aneurysms. Am J Pathol 2002 Nov;161(5):1701-10.
(8) Touat Z, Ollivier V, Day J, Michel JB. Renewal of mural thrombus releases plasma markers and is involved in aortic abdominal aneurysm evolution. Am J Pathol 2006;in press.
(9) Schouten O, van Laanen JHH, Boersma E, Vidakovic R, Feringa HHH, Dunkelgrün M et al. Statins are Associated with a Reduced Infrarenal Abdominal Aortic Aneurysm Growth. Eur J Vasc Endovasc Surg 2006;32:21-6.
(10) Hackam DG, Thiruchelvam D, Redelmeier DA. Angiotensin-converting enzyme inhibitors and aortic rupture: a population-based case-control study. Lancet 2006;368:659-65.
(11) Lindholt JS. Relatively high pulmonary and cardiovascular mortality rates in screening-detected aneurysmal patients without previous hospital admissions. Eur J Vasc Endovasc Surg 2007 January;33(1):94-9.
(12) Diehm C, Kareem S, Lawall H. Epidemiology of peripheral arterial disease. Vasa 2004 November;33(4):183-9.
(13) McDermott MM. The magnitude of the problem of peripheral arterial disease: epidemiology and clinical significance. Cleve Clin J Med 2006 Oct;73 Suppl 4:S2-S7.
(14) Norgren L, Hiatt WR, Dormandy JA, Nehler MR, Harris KA, Fowkes FG et al. Inter-Society Consensus for the Management of Peripheral Arterial Disease (TASC II). Eur J Vasc Endovasc Surg 2007;33 Suppl 1:S1-75.
(15) Diehm C, Kareem S, Lawall H. Epidemiology of peripheral arterial disease. Vasa 2004 Nov;33(4):183-9.

Machine Translated by Google

(16) McDermott MM. The magnitude of the problem of peripheral arterial disease: epidemiology and clinical significance. Cleve Clin J Med 2006 Oct;73 Suppl 4:S2-S7.
(17) Diehm C, Kareem S, Lawall H. Epidemiology of peripheral arterial disease. Vasa 2004 Nov;33(4):183-9.
(18) McDermott MM. The magnitude of the problem of peripheral arterial disease: epidemiology and clinical significance. Cleve Clin J Med 2006 Oct;73 Suppl 4:S2-S7.
(19) Graham I. European guidelines on cardiovascular disease prevention in clinical practice: Executive summary. Atherosclerosis 2007 Sep;194(1):1-45.
(20) Hurst NP, Ruta DA, Kind P. Comparison of the MOS short form-12 (SF12) health status questionnaire with the
SF36 in patients with rheumatoid arthritis. Br J Rheumatol 1998 Aug;37(8):862-9.
(21) Hurst NP, Ruta DA, Kind P. Comparison of the MOS short form-12 (SF12) health status questionnaire with the
SF36 in patients with rheumatoid arthritis. Br J Rheumatol 1998 Aug;37(8):862-9.
(22) Graham I. European guidelines on cardiovascular disease prevention in clinical practice: Executive summary. Atherosclerosis 2007 Sep;194(1):1-45.
(23) Hurst NP, Ruta DA, Kind P. Comparison of the MOS short form-12 (SF12) health status questionnaire with the SF36 in patients with rheumatoid arthritis. Br J Rheumatol 1998 Aug;37(8):862-9.

Machine Translated by Google

Appendix 1. Preventive Cardiovascular Examination for Men Aged 65–74 in the Central Jutland Region

Participant Information

We would like to ask if you would participate in a scientific population study investigating the preventive effect of ultrasound examination on the consequences of abdominal aortic enlargement and blood circulation in the legs in men aged 65–74 years.

Since enlargement of the abdominal aorta can lead to serious and potentially life-threatening circulatory problems, it is important to detect such enlargement at an early stage, that is, before any symptoms occur. In cases of larger enlargements, surgery is recommended due to the risk of rupture. In cases of smaller enlargements, regular monitoring is recommended to prevent further expansion. Examination for enlargement of the abdominal aorta is performed using ultrasound scanning, which is a quick procedure without discomfort or side effects.

Early atherosclerosis in the legs, which is usually a benign condition, increases the risk of blood clots in the heart and brain, as there may also be atherosclerosis in the arteries there. Atherosclerosis and the associated risk of blood clots can be prevented with medication and lifestyle changes. Atherosclerosis in the legs is assessed by measuring the ankle blood pressure using ultrasound and comparing it to the blood pressure measured in the arm.
If there are signs of atherosclerosis in the legs or enlargement of the abdominal aorta, preventive measures as mentioned above will be recommended, and a blood sample will be taken to measure cholesterol.

We would therefore like to invite you to an ultrasound and cardiovascular examination on **[date] / 2008, at [time]** at the Medical Outpatient Clinic, located in the reception area and Emergency Department at Viborg Regional Hospital. Signs mark "CARDIOVASCULAR EXAMINATION" will make it easy to find. It would be helpful if you wear footwear that can be easily removed. The examination takes approximately 10 minutes and is without discomfort or side effects.

Upon arrival, you will receive more detailed oral information. You will then have the opportunity to take time to consider whether to participate. Participation in the examination is voluntary, and you are welcome to bring a companion. As a study participant, you have certain rights, which you can read about in the attached supplement from the Central Danish Scientific Ethics Committee.

The study is supported by the EU and the Danish Heart Foundation, but unfortunately not enough to cover transportation costs or provide assistance with travel.
As part of the project, we will ask you to answer some questions in the attached questionnaire. This information will be treated confidentially and used solely for scientific purposes. The collected information and findings will contribute to a larger scientific assessment of the effectiveness of the preventive program.
If you have any questions or concerns regarding the examination, please contact us—preferably using the attached reply form or via email.

Kind regards,

Chief Researcher Jes S. Lindholt, Vascular Surgery Department, Viborg Regional Hospital
E-Mail: Forskningssektionen@Sygehusviborg.dk | Phone: +45 8927 2447

Machine Translated by Google

Feedback.

Regarding preventive circulatory examination

Tick.

1. I do not want the survey: ___
2. I want the examination, but cannot come that day: ___
3. I want the examination, but cannot come at the offered time: ___

Notes:____

____

____

CPR NUMBER ____

NAME ____

ADDRESS ____

TEL ____

Send to Research Consultant Jes Lindholt, Research Section, Viborg Regional Hospital, PO Box 130, 8800 Viborg.

Alternatively via e-mail: forskningssektionen@sygehusviborg.dk or by telephone 89272447

The rights of the subject in a biomedical research project.

As a participant in a biomedical research project, you should know that:

- Your participation in the research project is completely voluntary and can only take place after you have received both written and oral information about the research project and signed the consent form.
- You may withdraw your consent to participate and withdraw from the research project at any time, verbally, in writing or by other clear statement. If you withdraw your consent, this will not affect your right to current or future treatment or any other rights you may have.
- you have the right to bring a family member, friend or acquaintance to the information interview
- you have the right to a period of reflection before signing the consent declaration
- information about your health conditions, other purely private information, team and other confidential information about you that arises in connection with the research project is subject to a duty of confidentiality
- Storage of information about you, including information in your blood samples and tissues, is carried out in accordance with the rules of the Personal Data Processing Act and the Health Act.
- It is possible to gain access to trial protocols in accordance with the provisions of the Public Access Act. This means that you can gain access to view all documents relating to your participation in the trial, except for those parts that contain trade secrets or confidential information about others.
- it is possible to complain and receive compensation according to the rules of the Act on Access to Complaints and Compensation within the Health Service

(This supplement is published by the Central Scientific Ethics Committee and can be attached to the written information about the biomedical research project. Questions about a project should be directed to the regional committee that has approved the project)

Machine Translated by Google

Appendix 2. Questionnaire in connection with the preventive circulatory examination of 65-74 year old men in the Central Jutland region

Social Security Number:____________________________ **Name:**____

Question 1a: Have any of your close relatives had enlargement of the abdominal aorta?

No: __, Yes:___, if yes - who (tick – preferably more than one) Father: __ Mother: __Brother:__ Sister:___

Question 1b: Have any of your close relatives had poor circulation in the legs (not varicose veins)?

No: __, Yes:___, if yes - who (tick – preferably more than one) Father: __ Mother: __Brother:__ Sister:___

Q. 1c: Have any of your close relatives had high blood pressure?

No: __, Yes:___, if yes - who (tick – preferably more than one) Father: __ Mother: __Brother:__ Sister:___

Question 2. Do you get pain in one or both legs when you go for a walk (please tick)? No:___ Yes: ___

If yes, please answer the following questions: 2A. How far

can you go before you have to stop? ________ meters

2B. Where is the pain located (check more than one place if necessary)? Back: ____ Buttock:____ Hip:____ Thigh:____ Knee: ____ Lower leg:____Foot: ____

2C. Which leg is it (please tick): Right:____ Left: ____Both: ____

2D: Does the pain go away quickly at rest? No:___ Yes: ___

Question 3: What is your height?: _______cm, and weight?: _______kg

Question 4. Tobacco consumption (check box).

Never smoked:____ Formerly smoked:_____ Current smoker: ____

Question 5. Do you have diabetes (please tick)?: No:___ Yes: ___

If so, how long have they had it (specify number of years)? _______ year,

and how is it treated (tick)? Diet only: ___ Tablet:___ Insulin:___

Question 6. Do they have high blood pressure (check)?: No:___ Yes: ___

If yes, how is it treated (check)? None: ___Diet only: ___ Tablet:___

Question 7. Do they take fish oil as a dietary supplement (please tick)? No: ____ Yes: ____

Question 8. Medication use (state the preparation and dosage of what you are using or bring a note).

Question 9. Quality of life and well-being. The following five statements are about how you have been feeling over

the past 14 days. Please tick the box that best applies.

	How much of the time does the statement fit?					
	all the time	most of the time	a little over 50% of the time	a little less than 50% of the time	some of the time	at no time
I am in a good mood.	☐	P P	P	P P	P P	P P
I feel energetic and active.	P		P			
My everyday life is filled with things, which interests me	☐	P	P	P	P	P
When I wake up, I feel fresh and rested.	P P	P P	P P	[illegible]	P P	☐
I feel calm and relaxed				[illegible]		P
Scoring	nx20+ ☐	nx16+ ☐	n x 12 ☐ +	nx8+ ☐	n x 4 ☐ +	n x 0 ☐
In total						P

Appendix 3. Consent to participate in the trial

THE CENTRAL SCIENTIFIC ETHICS COMMITTEE

The Danish National Committee on Biomedical Research Ethics

Informed consent to participate in a biomedical research project.

Title of the research project: Preventive circulatory examination of 65-74 year old men in the Central Jutland region

Statement from the subject:

I have received written and oral information and I know enough about the purpose, method, advantages and disadvantages to agree to participate.

I know that participation is voluntary and that I can always withdraw my consent without losing my current or future rights to treatment.

I consent to participate in the research project and have been given a copy of this consent form as well as a copy of the written information about the project for my own use.

Subject's name: ____

Date: ______________ Signature: ____________________________________

Statement from the person responsible for the trial:

I declare that oral information about the project has been provided, written information has been provided, and that consent has been given for the subject to participate.

Name of the person responsible for the study: ____

Date: ______________ Signature: ____________________________________

Machine Translated by Google

Appendix 4. Written information about data registration and biobanking

Research section

Viborg Regional Hospital

Participant information regarding clinical database and biobank for carotid artery occlusion and impaired circulation in the legs

We would like to ask you to participate in a scientific registration of some health information about you with an associated blood test.
In connection with developing our abilities to treat carotid artery dilation and mild atherosclerosis in the legs, we would like permission to register health information about you in a database and take a blood sample.

The health information corresponds to that you have provided and the findings made during the ultrasound scan and blood pressure measurement, so you will not be examined further and will not be exposed to any additional risks and inconveniences beyond those involved in taking the blood sample. This is a "regular" blood sample taken from the bend of the elbow, which may cause a momentary discomfort and a subsequent bruise.

The information and blood samples will only be used for scientific purposes that will improve our ability to treat coronary artery disease or atherosclerosis in the legs.
The information regarding the study will be collected in a register, where Research Supervisor Jes Lindholt will be the only person with access to the information, where the CPR number will be recoded so that no one can see it and know that it is your information. You can access your own data at any time.

The use of the blood samples for later scientific studies will only take place after approval by the scientific ethics committee.

It is of course voluntary whether you want to participate, and it will not affect your future check-ups and treatment if you do not participate.

To ensure that no experiments are conducted on humans or sensitive health information is recorded without the consent of the person concerned, the law requires that you sign a statement that you have been informed both verbally and in writing and that you agree to participate in the study.

Kind regards

Research senior
physician Jes S. Lindholt

Research Section, Viborg Regional Hospital
Tel.: 8927 2447

Machine Translated by Google

Appendix 5. **Information on preventing atherosclerosis**

What is atherosclerosis?

Atherosclerosis is the build-up of calcium and fat in the blood vessels. The deposits stiffen the blood vessels, reduce blood flow and, in the worst case, can cut off the blood supply, causing a blood clot.

Why do people get atherosclerosis?

It is not known exactly why people develop atherosclerosis, but there are a number of risk factors that increase the degree of atherosclerosis. These include smoking, being overweight, high blood pressure, and high cholesterol. and poorly controlled diabetes.

What can you do yourself?

By adjusting your lifestyle, you can prevent atherosclerosis from worsening. As a rule, you will achieve that the problems you have had become less or disappear completely. Atherosclerosis in the legs is a sign of possible atherosclerosis in other arteries of the body, including the heart. This means that changing your lifestyle will also prevent the risk of heart disease.

Smoking cessation

The most important thing is to stop smoking. There are many aids and courses for smoking cessation.
Quitting smoking helps so many that about half of those who seek medical attention for pain due to atherosclerosis are so satisfied that they do not want further treatment.
The weight will increase a little, but don't overreact. A few extra kilos are nothing compared to the benefits you get from quitting smoking. Avoid dieting, as they increase your desire to smoke. Instead, eat extra healthy food. Be active, go for walks, or start cycling.
There are useful tips at *www.hjerteforeningen.dk* and *www.doktorstop.dk*

The diet

You should look at your diet and eat less fat and more vegetables. Many good books have been published on the subject. We can recommend a diet consisting of lots of bread, fruit, vegetables, small amounts of lean meat, fish and rapeseed oil. If you are overweight, it is important to lose weight - a changed diet is important for sustained weight loss.

A single glass of red wine a day is good, but you should limit yourself to beer and you should not drink more than the Danish Health Authority recommends: a maximum of 14 units per week for women and a maximum of 21 units per week for men.

If you have diabetes, it is important that it is well-regulated.

Exercise

Finally, it is important to get more exercise.
Walking is the best thing to do if you have leg pain due to atherosclerosis. This is because it stimulates the formation of new small blood vessels. Three hours of walking a week will help so much that they

Most people are satisfied with how far they can walk. Start with short walks of about 20 minutes a day, and walk until you feel pain in your legs (claudication). Take a break and continue. Exercise is also beneficial for your heart, weight and “cholesterol levels”.

What can we offer you?

Treatment of high cholesterol

Elevated cholesterol can be lowered by changing your diet (possibly dieting), exercise and medication.
Cholesterol is one of the body's fats (lipids). Cholesterol and another fat, called triglyceride, are important building blocks in all of the body's organs.
The cholesterol content in the blood depends on the diet and the body's own production and "handling" of cholesterol.
Furthermore, the tendency to the common type of elevated cholesterol is probably hereditary.
High cholesterol levels can contribute to the narrowing of the arteries – called “atherosclerosis”. Atherosclerosis occurs when cholesterol is deposited in the walls of the arteries. A better name would therefore be “atherosclerosis”.

By lowering the cholesterol content in the blood, atherosclerosis can be slowed down, perhaps reduced - but not eliminated if atherosclerosis has already occurred.
A very important thing you can do yourself is to change your eating habits. Fat intake should be reduced, and the fat you do have to eat should be of the right kind. In general, animal fat, i.e. from dairy products, eggs and animals, should be limited as much as possible.
When signs of incipient atherosclerosis are found, dietary changes are rarely effective. By determining your cholesterol level, you can quickly determine this. Approximately 90% should be on additional cholesterol-lowering treatment.
The most commonly used is Simvastatin, which is the most proven and has been shown to reduce the likelihood of blood clots by 25%. The medication rarely has side effects, although most often temporary muscle pain.
If symptoms persist, you can switch to another preparation with good effects.
The treatment is lifelong – so you must go to your doctor for prescription renewal.

Blood thinning treatment

When arteries become narrowed, the risk of blood clotting in the narrowing increases, causing a blood clot to form.
This occurs when the platelets in the blood stick together. This ability can be inhibited with a very low dose of codimagnyl – just 75 mg per day. It has been shown to reduce the risk of blood clots by 15%.
Regular codimagnyl is 500 mg, so originally children's magnyl was used, but now they are available in many versions, such as hjertmagnyl and hjerdyl. They are available over the counter. Side effects can be "too much stomach acid", stomach pain or allergies. If necessary, you should contact your own doctor.
The treatment is lifelong.

Kind regards

Senior Research Physician, Ph.D.
Jes S. Lindholt

Research Section, Viborg Regional Hospital

Machine Translated by Google

Appendix 6.

Supplement regarding the collection for the purpose of storing biological material in a research biobank.

Clinical database and biobank for carotid artery occlusion and impaired circulation in the legs

I consent to participate in the research project and to my biological material being collected for storage in a research biobank. I have been given a copy of this consent form and a copy of the written information about the project for my own use.

Date: ________________________ Signature: ________

According to the law, you must be notified if new, significant information about your health conditions emerges during a research project that is not currently known. However, the law also states that you can refuse to receive this information. This may, for example, concern a predisposition to a hereditary disease that may or may not manifest itself after a number of years. However, it may also concern other health information that only emerges in connection with the implementation of the research project.

If you would like to opt out of receiving information about any new health information that may emerge during the research project, please tick here:

I hereby decline to receive information about any new health information: (place x)

Date: ________________________ Signature: ________

Machine Translated by Google

SHORT CURRICULUM VITAE

CPR: 170763-0031

Research Associate Professor, Senior Consultant in Vascular Surgery, Ph.D, FEBVS

Jes Sanddal Lindholt

Nørremøllevej 101, 8800 Viborg
Tel.:35397717 or 22440000

Email: jes.s.lindholt@sygehusviborg.dk

Website: www.sygehusviborg.dk/sw53562.asp

Chronology

1990. Medical degree. Aarhus University. 6½ years.
1998. PhD degree at the Faculty of Health Sciences, Aarhus University: "Considerations and experiences of screening for abdominal aortic aneurysms". Fadl Forlag.
2000. Board member of the Danish Society of Vascular Surgery
2003. Specialist in vascular surgery after training in Skejby, Viborg and Rigshospitalet.
2004. Associate professor at Aarhus University and research physician at RH Viborg
2004. Co-founder of the European research consortium: FAD (Fighting Aneurysmal Disease)
2005. Editorial Board member of the European Journal of Vascular and Endovascular Surgery
2006. Course leader and lecturer at Aarhus University's research course for future specialist doctors
2006. Danish vascular surgery representative in the Union Européenne de Medecins Spécialists (UEMS)
2007. Chairman of the Research Committee at RH Viborg

Summation of scientific publications

Books: 2
Book chapters: 4
Peer reviewed overview articles: 15
Peer reviewed original publications: 75
Invited speaker at scientific societies: 26
Scientific lectures after peer review: 66
Scientific awards: 7

Reviewing activity

Reviewer for the Lancet (currently 4 times)
Reviewer for the British Medical Journal (currently 3 times)
Reviewer for The Circulation (currently 3 times)
In addition, for The Netherlands Health Science Foundation, The Scandinavian Journal of Infectious Diseases The National Medical Research Council (MRC), United Kingdom, Journal of Vascular Surgery, European Journal of Vascular and Endovascular Surgery, European Journal of Cardiovascular Prevention and Rehabilitation, BMC Public Health,r International Archives of Allergy and Immunology, external referee for the Netherlands Organization for Scientific Research (NOW), Atherosclerosis, Thrombosis and Vascular Biology, and Br J Surg

International research relations

1. Member of the European research consortium consisting of 15 research institutes and laboratories in Europe incl. Iceland and Turkey. Responsible for joint web based database.

Machine Translated by Google

2. Member of The Collaborative Aneurysm Screening Study (CASS) Group consisting of representatives from the existing randomized screening studies regarding AAA
3. Principle investigator in a Nordic randomized multicenter study with 12 participating centers regarding permanently heparinized artificial blood vessels
4. Research collaboration with senior researcher GP Shi's research group at Harvard University, Boston, USA

Scientific Ph.D. supervision:

Completed Ph.D. projects

1. Project supervisor for *Sten Vammen.* "Prevention and treatment of abdominal aortic aneurysms", Aarhus University (1997-2000).

2. Project supervisor for *Jette B. Støvring.* "A randomized double blinded interventional trial of roxithromycin versus placebo as secondary and tertiary prevention of lower limb atherosclerosis". Aarhus University (2002-2005)

3. Project supervisor for *Sigitas Urbonavicius.* "Identification of peptides and proteins predictive for the natural history of abdominal aortic aneurysms" Aarhus University (2004-8).
In collaboration with the Department of Biochemistry, Aarhus University

In ongoing Ph.D. projects

5. Supervisor for *Ljubica Andersen.* “Acute upper limb ischemia and atrial fibrillation” (2005-).
In collaboration with the Medical Department, RH Silkeborg.

6. Main supervisor for *Anne Mette Momsen:* Caffeine and Intermittent Claudication (2006-).
In collaboration with the surgical research unit, Herning Central Hospital.

7. Principal supervisor *Anette Høgh.* The importance of ACE inhibitors and beta-blockers for vascular surgical reconstructions and amputations (2006-).
In collaboration with the Department of Clinical Epidemiology, Aarhus University Hospital

8. Principal supervisor for PhD student *Grazina Urbonaviciene.* “Clinical prospective study of potential new cardiovascular risk factors” (2007-).
In collaboration with the Medical Department, Aarhus Municipal Hospital

11. Main supervisor for PhD student *Casper Nielsen (2008-).* “D-Lactate as a marker for intestinal ischemia – an experimental study on pigs”.
In collaboration with Gastrosurgery Department L, Aarhus Municipal Hospital

Ratings

PhD thesis by Tryfon Vainas, University of Maastricht, Netherlands. 2006
Censor for The European Board of Vascular Surgery, Prague. 2006
PhD thesis by Gustav Petersen, University of Bergen, Norway. 2007
Censor for The European Board of Vascular Surgery, Madrid. 2007
Additionally, external examiner for 5 master's theses

Accumulated research support …………………………………….……….……... 20,702,135
