# Supplementary material for: Association of urate-lowering therapies with abdominal aortic aneurysm growth and clinical events in men: A population-based cohort study
Source: PLoS One. 2026 Jul 31;21(7):e0341242. doi: 10.1371/journal.pone.0341242 (PMC13427005; doi:10.1371/journal.pone.0341242)
Supplement: S6 File — (PDF) [file pone.0341242.s006.pdf]

The Danish Cardiovascular Screening (DANCAVAS) Trial
- A combined, multicentre randomised controlled intervention trial and cohort study

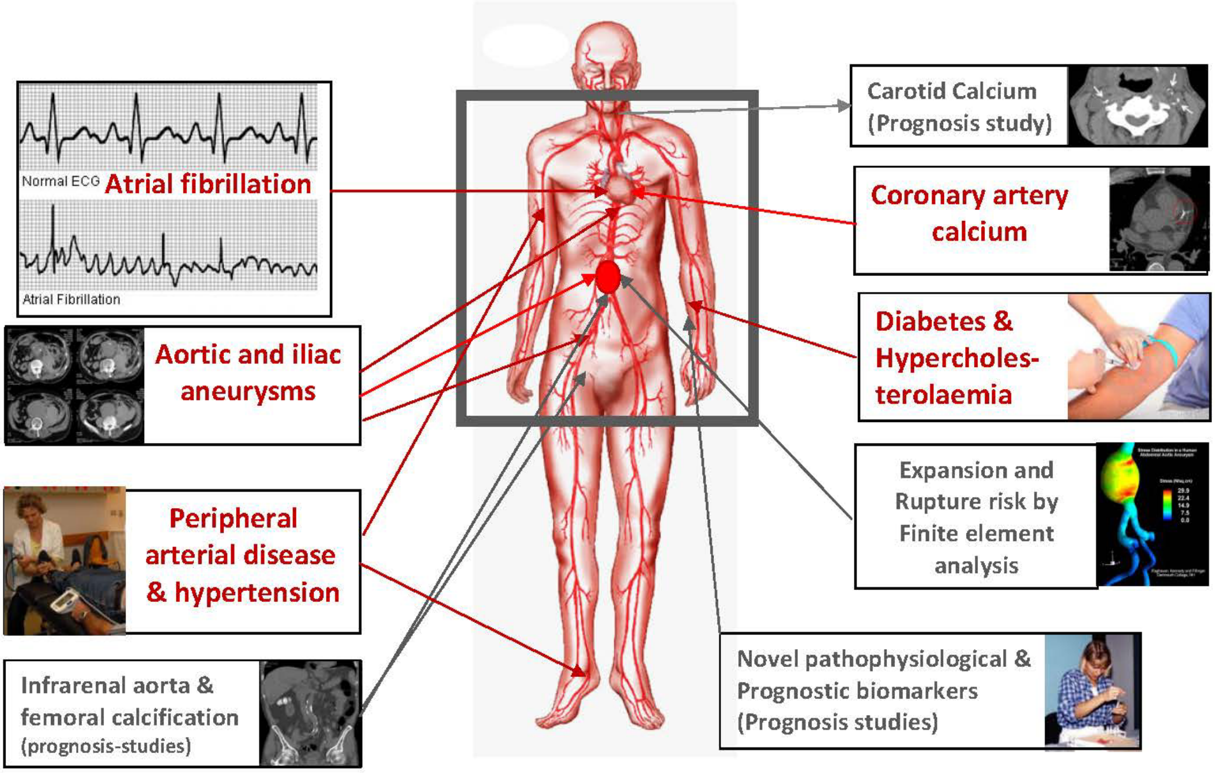

Executive committe

Assoc. professor, Consultant in Cardiology, PhD, ***Axel Diederichsen***(1,2)
Head of CIMA, Professor in Biochemistry, Consultant, dr.med., ***Lars Melholt Rasmussen*** (1,3)
Professor of Vascular Surgery (1,4), Consultant, dr.med. PhD ***Jes S. Lindholt***

1. Elite Research Centre of Individualised Medicine in Arterial Diseases (CIMA), Odense University Hospital
2. Department of Cardiology, Odense University Hospital
3. Department of Biochemistry and Pharmacology, Odense University Hospital
4. Department of Cardiothoracic and Vascular Surgery, Odense University Hospital

CIMA
Centre for Individualized Medicine in Arterial Diseases

Lay Description:
The DANCAVAS study will for the first time test the impact of advanced cardiovascular examinations on future cardiovascular events in the general public. The expected potential effect is the prevention of up to 1,000 deaths and a significantly greater number of hospital admissions in Denmark.
The significant increase in the average life span increases the frequency and the societal challenge of managing serious age-related diseases, especially cancer and cardiovascular diseases. A routine check by a general practitioner is not sufficient to detect incipient cardiovascular disease. Therefore, the main objective of this research project is to investigate whether advanced cardiovascular screening will prevent cardiovascular events, and whether the possible health benefit is cost effective.
Half of the 30,000 randomly selected 65-74-year-old men will be invited to an advanced cardiovascular preventive screening at 4 screening sites in 2014-2015. The screening will include the following: (1) a CT scan of the major arteries from the neck down to the legs to detect coronary calcification and aneurysms, (2) blood pressure measurements on the upper arms and ankles to detect peripheral arterial disease, (3) an assessment of the heart rhythm, and (4) a measurement of the cholesterol and blood sugar levels. We expect to demonstrate at a minimum significant calcification of the coronaries in half of the participants, atherosclerosis in the legs in 1 out of 10, and dilated arteries in one out of 20. In the case of positive findings, preventive actions, including medical treatment and possibly surgery, will be taken. Biological sampling will be performed for biomarker and translational research.
Registered follow-up on hospitalisations, death due to cardiovascular disease, and health economic evaluations will be performed at 3, 5 and 10 years to assess possible health and/or societal benefits of the screening. Possible psychological consequences of the screening will be evaluated as well.

Introduction
Although cardiovascular diseases (CVD) have decreased during the last two decades, CVDs are still some of the most predominant causes of morbidity and mortality in the western world, including Denmark, where approximately 420,000 people have recognised symptoms.(1;2) Due to an aging population, the decline in CVD incidence observed during the past decades has not led to a decrease in hospital admissions and health-related costs due to CVDs. In Denmark, approximately 14,000 people die annually from CVDs, compared to 16,000 deaths caused by cancer, the most common cause of death. The cost of hospital admissions is DKK 4.6 billion and the medicines cost an additional DKK 2.4 billion. In addition, an unknown number of visits to the general practitioner occur.
Screening of CVD has been discussed intensively.(3;4) Ultrasound-based screening for abdominal aortic aneurysm (AAA) seems to reduce mortality,(5) while evidence supporting screening for ischemic heart disease is lacking. Population-based screening of higher risk individuals with the intention to initiate preventive treatment has not been associated with a reduction in all-cause or cardiovascular mortality.(6;7)
To assess the risk and target preventive interventions, global risk scores, such as the HeartScore and Framingham, are recommended. These scores are useful for combining individual risk factors (age, gender, diabetes, cigarette smoking, blood pressure, and cholesterol levels) into a single quantitative estimate. In Europe, individuals with a 10-year risk of CVD death ≥5% qualify for primary prevention, including lifestyle intervention and in some cases, drug treatment. In individuals with a CVD death risk ≥10%, drug treatment is more frequently required. However, in individuals older than 60 years, the risk scores should be interpreted more leniently due to a lack of evidence.(8;9) Guideline recommendations on interventions for the elderly population are lacking despite the fact that more than 95% of CVD deaths occur in people above 65 years old.
Consequently, the primary aim of this unique population-based, randomised trial will be to evaluate the benefits and cost-effectiveness of using non-contrast full body CT scans (to measure coronary artery calcification (CAC) and identify aneurysms) and measurements of the ankle brachial blood

pressure index (ABI) as part of a multifocal screening and intervention programme for CVD in men aged 65-74. Secondary aims will be the prospective observational studies of the prognostic importance of the calcification scores of the carotid, aorta, iliac, and femoral arteries, as well as the pathophysiological and translational biomarker studies that are made possible by biobanking.

Calcified arteries: carotid, coronary, aortic, iliac, and femoral arteries
Calcifications of any artery can be visualised by a CT scan without contrast. This technology was especially developed to evaluate for CAC, and several studies have demonstrated that the CAC scores improve the discrimination and reclassification of CADs above and beyond the traditional risk factors.(10-14) The prognostic value of grading calcifications of the carotid, aortic, iliac, and common femoral arteries is unknown. A few randomised studies have insufficiently evaluated the effect of statin treatment in asymptomatic patients with CAC.(15;16)

Aneurysms
Level A evidence has shown that ultrasound-based screening for AAA in men aged 65-74 years in Denmark reduces AAA mortality by 66% at a cost of DKK 1,500-5,500 per life-year gained,(5;17) and the procedure is being implemented in the UK, USA and Sweden. By extending the screening to include a non-contrast abdominal CT scan for CAC, the screening for AAA will incur a small additional cost, but it will also uncover thoracic aortic aneurysms (TAA) and iliac aneurysms (IA). Modern endovascular treatment may provide a low risk intervention for these aneurisms. AAA, TAA, and IA will be diagnosed with 100% sensitivity and specificity by the extended use of a non-contrast CT scan.

Peripheral Arterial Disease (PAD)
International scientific studies indicate that approximately 5-10% of men above 60 years old show signs of PAD and the proportion increases with age. Although 78% of the patients are asymptomatic, approximately 25-30% of the patients with PAD will die from CVD within a 5-year period regardless of their presentation of symptoms. An even higher proportion will need hospitalisation due to their CVD.(18) Efficient prevention of CVD specific deaths, amputations and other CVD events can be achieved through smoking cessation, exercise, a healthy diet, aspirin, lipid-lowering treatment, and treatment of elevated blood pressure.(19;20)

Hypothesis
The primary hypothesis is that the offer of an extensive circulatory screening and intervention programme fulfils the WHO criteria for screening,(21) especially concerning the significance of the diseases, the treatment benefits, and the cost effectiveness.

Objectives
The primary objective of the study is to establish the effect and cost-effectiveness of an extensive circulatory screening and intervention programme for men aged 65-74 years in a randomised controlled trial.

Design
This is a multicentre randomised controlled interventional trial, randomising half of the study participants to the usual care without any screening (*control group*), while the other half of participants are invited to a screening and intervention programme that measures traditional risk factors, CAC, aneurysms, and PAD (*screening group*). The control group will not be informed about the trial.

Material and Methods
Power calculations

A total of 30,000 (2x14647) men are needed to detect a 5% reduction in overall mortality (HR=0.95) with the risk for a type 1 error=5% and the risk for a type II error=80%. We expect a two year enrolment, a 10 year follow-up, and a median survival of 15 years among the controls. The randomisation ratio will be 1:1 with an HR=0.95.(22) The recruitment rate is estimated to be 70%.(5;10)

Randomisation
In blocks of 1000 individuals, the Civil Registration Number, name, and address information will be supplied by the Patient Administrative System. Randomisation will be performed in Epidata by providing each individual a random number from 1-100. Those numbered 51+ will be invited to participate in the screening program.

Inclusion and exclusion criteria
The inclusion criteria are male gender, age between 65 and 74 years, and living in the involved communities. There are no exclusion criteria. (See Figure 1)

Screening
The central project secretariat will invite the selected individuals, and the invited individuals can either phone or email their interest to join the trial. A small questionnaire on life style, medical history, and the QoL a.o. will be enclosed with the invitation. Non-responders are re-invited once. The participant will be informed at attendance to the screening visit, and their consent will be obtained together with the questionnaire, weight, height, and waist circumference. The HbA1c and lipid parameters will also be measured. Biobank blood samples are then taken, centrifuged, labelled, cooled, and stored at -80 degrees Celsius. Bilateral blood pressure will be recorded three times after 5 minutes of supine rest. If any of the peripheral pulses are missing, the ankle blood pressure will be measured concurrently with the brachial blood pressure (VIVA data will be submitted for publication). The CT scan will cover the area from the mandibular bone distally to the proximal third of the femur. Calcium scores for the common carotids, coronary arteries, aorta, and common iliac, and femoral arteries will be calculated. The aorta will be visualised, and in the case of dilation, the maximal perpendicular outer to outer AP diameter will be measured. If no dilation is found, the AP diameter will be measured just above the bifurcation. All of the results will be sent to the central project secretariat, where the data will be entered into the project database.

Follow-up visit after screening
For all screening locations, if the CAC is above the median[1] or if TAA/AAA/IA (definition: diameter ≥ 35, 30 and 25 mm, respectively) or PAD (Def.: ABI ≤ 0.9 or > 1.4) is detected, "the patient" will be informed at a follow-up visit of the finding and its implications. At this visit, the patient will be recommended suitable prophylactic measures, including smoking cessation, walking/exercise, a low-fat diet, and starting treatment with aspirin 75 mg/day and atorvastatin 80 mg/day. In patients with AAA/IA, the aorta will be reassessed by ultrasound, and the popliteal arterial segments will be screened for an aneurysm (defined>15 mm). If the TAA/AAA/IA or popliteal aneurysm exceeds a diameter of ≥ 60, 50, 35 or 20 mm, respectively, the patient will be referred for a vascular surgical assessment at one of the three involved vascular centres that use a uniform size threshold for the repair of asymptomatic aneurysms (65, 55, 40 or 25 mm, respectively).(23) An annual check-up including a CT scan of the TAA or ultrasound scans of the AAA or IA will be offered.
If no positive findings (CAC above the median, aneurysm or PAD) are detected, the participants will be informed of the findings by e-mail or ordinary post as preferred.
Independent of the above findings, the patients will be encouraged to see their GP for further assessment if potential undiagnosed hypertension (systolic blood pressure >160 mmHg),(24)

---

[1] http://www.mesa-nhlbi.org/Calcium/input.aspx

diabetes mellitus (HbA_{1c} >48 mmol/mol),(25) or significant isolated hypercholesterolemia (total-cholesterol >8.0 mmol/l) are observed, as possible continuous medical treatments will be better managed by the GPs. The GPs will be informed by a letter of all negative and positive results and the initiated actions.

Annual follow-up

Annual follow-up will be conducted through data extraction from registries and the life-style questionnaires that will be sent to the participants. Prescription records that show the implementation of pharmacological interventions, as well as hospitalisation records and disease specific mortality data, will be collected from the government maintained nationwide registries, including all Danes, as no delivery of prescribed medications, hospitalisations and deaths is possible in Denmark without being recorded in these registries. These individual-based data are available for researchers. Consequently, these registries have been continuously subjected to an in depth validation process and have been proven valid regarding data on CVD and mortality.(26-32) Aneurysms that do not exceed the size-threshold for a vascular surgical evaluation referral will be offered either an annual CT (TAA) or US scan (AAA, IA) depending on the location of the aneurysm. The attendance rate for these control scans are documented in the RCTs performed previously in Denmark to be 95%.

Pilot studies and quality control studies

The age specific median for the CAC score will be established after the first 500 examinations. Non-contrast CT and ultrasound will be performed in the first 500 men, and using ultrasound as the gold standard, the concordance between non-contrast CT and ultrasound in the detection of AAA will be analysed.

The screening method for hypertension (after 5 minutes of supine rest) will be validated in the first 500 men with blood pressure measurements of patients in a seated position in an isolated room as the gold standard. In both cases, the blood pressure will be measured three times. In addition, 24 hour measurements will be taken in a subgroup.

Supervised measurements of the CAC scoring, the diameters of thoracoabdominal aorta and iliac arteries, and the ABI will be performed by senior examiners in the first 50 cases, followed by interobserver validation in the next 50 cases. If the mean differences in the CAC score, aortic diameter, and ABI are below 10%, 2 mm, and 15%, respectively, unsupervised examination will be allowed; otherwise, the training and validation procedure will be repeated. After the first 500 examinations, re-validation of the interobserver agreement will be repeated.

Personal contact information, the randomisation process, the central secretary administration, staff education, the acceptance rates to the screening invitation, the standardisation of screening and treatment protocols, the systems for collecting initial and follow-up data, the response rates to the questionnaires, quality control of the procedures, and the organisation of trial data will be tested and evaluated in the first 2,000 male residents of Odense recruited into the study.

A pilot study involving 1,000 randomly selected women aged 65-74 years will be performed and used in subsequent power calculations for women. This screening will be no different from the males in the main study.

Efficiency variables and statistical analyses

The entire study population, the controls as well as the screening group, will be monitored for a period of 10 years. The primary efficiency variable is overall mortality, while hospitalisations and deaths from cardiovascular diseases (cerebrovascular, cardiac, aneurysm, or other vascular) are the secondary variables. These endpoints are compared for the two groups using a Cox proportional hazards-regression analysis. The cost-efficiency calculation will be adjusted for the quality of life. An independent endpoint committee will review registry data on the causes of death and data from the Danish National Patient Register concerning hospital admissions. Supplemental data on

mortality and cardiovascular disease will be requested from hospitals and the GP if needed in validation of the national registries.

Cost effectiveness
The health economics of the screening program will be evaluated with two types of analyses. A trial-based evaluation will be conducted after 5 and 10 years of follow-up, whereas the lifetime perspective on the health economics of the screening will be evaluated in a separate decision analytic model for the men and women.

Financial and budgetary administration
The participating sites will fund the CT scans, room facilities, and the lipid and HbA_{1c} measurements, while the study organisation will cover the costs for the study staff and biobanking. The obtained funding will be administered by the Elitary Research Centre of Individualized Medicine in Arterial disease (CIMA), OUH, where the project secretary will be located. Expenses are primarily paid for by funds from public and private donations and sponsorships. The project managers have no economic affiliation to any of the foundations. Participants will not receive any remuneration or reimbursements for transportation costs.

Expected number of examinations, operations and visits
The four screening sites that will be established are in Svendborg, Odense, Vejle and Silkeborg. At each site, 37 men will be invited at ten-minute intervals for ten minutes long screening appointments on each of the days scheduled for screening with a total of 284 screening days. If each site can provide 2 weekly screening days, enrolment may be completed within one year. Individuals with positive findings will attend a 30 minute outpatient appointment for information and initiation of preventive actions instruction Three days a week is assumed to be needed for these outpatient appointments.
One in every 10 cases of AAA is expected to be referred for surgical assessment; among these referred cases, 90% are expected to undergo surgery. These surgical cases are estimated to add 1-2 AAA operations to the surgery schedule per month per department. The vascular surgery departments do not anticipate any problems with the handling of these cases due to the prevention of emergency cases.

Organisation
The Executive Committee, consisting of the cardiologist Axel Diederichsen, biochemist Lars M. Rasmussen, and the vascular surgeon Jes S. Lindholt, will handle the decisions regarding the administration, budget, overall organisation, data, use of biobanks, and principles for authorships.
The Steering Committee will consist of the members of the executive committee, Michael Hecht Olsen as the expert in cardiovascular risk factors, Jesper Hallas as the pharmaco-epidemiologist, a senior statistician who will be collaborating with Jesper Hallas, the health economist Rikke Søgaard, and one from each screening site.
All practical issues concerning the screening, follow-up, biobank and data sampling will be handled by the steering committee. In addition, the steering committee will participate as authors in the reporting of the primary endpoints.
The independent data monitoring committee (DMC) will consist of a statistician, a cardiologist, and a vascular surgeon to identify any suspected major safety effects. They will meet biannually for an update of events from the registries. The DMC will have the power to recommend termination of the study based on an evaluation of these results. The typical three reasons a DMC might recommend termination of the study are as follows: safety concerns, futility, and outstanding benefit. However, the latter will not cause termination of the study as the screening offer will generally not be offered, but an outstanding benefit will prompt a publication of the interim analysis.

The Advisory Board consists of the following international experts who have various special interests in specific areas: cardiologist Peter Libby (Brigham and Women's Hospital, Boston, MA, USA), cardiologist Raimund Erbel (West-German Heart Center Essen, Germany), statistician Simon Thompson (MRC statistical unit, Cambridge, UK), health economist Dorthe Gyrd-Hansen (University of Southern Denmark), translational CV researcher Jean B Michel (Hôpital Xavier Bichat, Paris, France), and translational AA researcher Guo Ping Shi (Brigham and Women's Hospital, Boston, USA).

Data registration
Data registration will be electronic and based on numeric codes established by the project secretary and kept in a locked room at CIMA. The database will be stored on an internal hospital drive to avoid the risk of data loss in case of a technical failure. Only the project secretary and the independent data review committee members will have access to the complete database. However, the executive and steering committees will have access to data concerning the invited group to secure follow-up and allow for observational studies.

Biobank
As a part of the study, a biobank will be organised. 40 mL of blood form each of the participants are centrifuged, labelled, and stored at -80 degrees Celsius. Analyses of (1) atherosclerosis and (2) inflammation in the vessel wall, (3) diseases in the myocardium, (4) calcium / phosphate metabolism and (4) proteomics are intended.
Remaining blood samples are planned to be stored for a ten-year period for future research, and subsequently data will be anonymous. This is approved by the Danish Data Protection Agency. Prior to future analyses approval by the Region Committee on Biomedical Research Ethics will be requested. The purpose of the biobank is to perform pathophysiological and translational studies, and to facilitate new knowledge of development and treatment of atherosclerosis.

Informed consent
Participants are mature men or females who are fully capable of deciding whether to accept or reject the invitation, and efforts have been made to provide a comprehensive explanation of the study in the invitation. The invitation to participate in the study is sent by mail to potential participants and in case of interest they are asked to contact the project secretariat by mail, e-mail, or phone. The participants are informed that they may bring a companion, preferably a spouse/partner, in connection with the examination. At attendance to the screening site and prior the examination, the participants are informed by study nurses. This interview takes place in a calm environment. Participants will be given time to consider their participation before deciding and are allowed one week of deliberation time. If the candidates wish to participate, they are asked to sign the written consent form.
In case of positive finding, participants will be offered an in-depth outpatient interview as soon as possible. At the consultation, information will be given concerning the prognosis of the positive finding and the need and benefit of prophylactic measures.

Ethical considerations
The medical examination will not cause any notable inconveniences and any definite risk. However, several large epidemiological studies do suggest that radiation exposure is associated with a slightly increased risk of cancer. The best studied cohort is the Japanese atomic bomb survivor cohort. In a group exposed to radiation doses of 5 – 100 mSv (a mean dose of 29 mSv), 4406 solid cancers were observed between 1958 and 1998, an excess of 81 solid cancers over the expected cancer rates. This finding corresponds to an excess relative risk of 2%.(33) No large studies involving medically exposed adult cohorts are available, but a linear no-threshold model has been considered. Thus, there may be no minimal radiation dose for an increased cancer risk, and the risk increases linearly with the radiation dose. The average dose in our pilot study was 1 mSv.(10) For comparison, a

typical dose of a mammogram is 0.2 mSv, the annual background radiation dose is 3 mSv, and the average annual limit for radiation workers is 20 mSv.(34) According to the Danish National Committee on Biomedical Research Ethics, a radiation dose of 0.1-1 mSv to subjects under 50 year of age is associated with an overall cancer risk in the magnitude of 1 in 100 000, and to subjects older than 50 years, the radiation dose can be increased by a factor of 5 to 10.
Screening offers are known to reduce the quality of life in the period leading up to the examination, but the effect fades out in the absence of a positive finding. It is unclear if a sustained reduction in the quality of life is caused by the diagnosis itself or by a comorbidity, but the reduction is modest. Such “secondary side-effects” should be weighed against the prophylactic benefits that might be achieved.
Statin treatment may increase the risk of diabetes mellitus (from 1.12% to 1.22% per year, adapted from (35)) while decreasing the risk of coronary and cerebrovascular events (from 1.29% to 1.00% pr year, and 0.55% to 0.45%, respectively, adapted from (6)). Aspirin increases the risk of haemorrhage (from 0.07% to 0.10% per year) while decreasing the risk of CVD (from 0.57% to 0.51% pr year).(36) These side effects should be weighed against the risk reduction achieved.
This is not a drug trial, since participants with incipient atherosclerosis are offered standard preventive medical treatment.
A more serious problem in connection with the detection of aneurysms is the risk of death caused by rupture of the aneurysm in conservatively treated cases and perioperative deaths in cases where the AAA might not have otherwise ruptured. This serious ethical problem has no solution at present. We hope that the studies with the planned biobank will facilitate the development of a prognostic model. A specific dilemma is the situations in which AAA is diagnosed and requires surgery that carries a 1-3% mortality risk, which should be weighed against the mortality risk of approximately 90% in AAA ruptures. When there is an AAA surgery indication, the patient is informed of the surgical risks and the risks of conservative treatment, and a surgery date is set. One of the project managers has experience in performing this function.
The project will be approved by the Southern Denmark Region Committee on Biomedical Research Ethics.

Publications
Project results reporting the primary end point will be published in peer reviewed international journals with AD and JL as the 1st and last authors, respectively. Positive as well as negative findings will be reported.

Strengths and Limitations
There are no exclusion criteria in the study, and this implies that patients with known CVD, such as former stroke and myocardial infarction, may also be invited to participate. This might be superfluous because these individuals have documented CVD, and preventive care should have already been established. However, these patients do have an increase prevalence of aneurysms and may therefore benefit from the screening.(37-39) This possible benefit will be explored in detail in subgroup analyses. Another limitation is the lack of a non-imaging experimental group. Thus, in case there are benefits to the screening, we will be unable to differentiate whether the benefit was due to the imaging findings or due to treatment of classical risk factors, such as hypertension. However, in many of these elderly subjects, primary medical intervention is controversial, and the decision is left to the individual subjects with the GP minimising the difference from the usual care. Additionally, in this study of males aged 65-74 years, the major problem of unexpected sudden deaths among young men is not addressed. However, as the population gets older, this study will provide important knowledge about initiating prevention among the retired men.

Feasibility
The two executive committee members Axel Diederichsen and Jes Lindholt have previously organised and implemented similar population studies concerning CAC, AAA, and PAD. Michael

Hecht Olsen has experience investigating risk factors in large clinical trials and population-based studies. Rikke Søgaard has experience in evaluating and modelling the cost effectiveness of screening programmes. Jesper Hallas is experienced in exploiting the unique Danish pharmaco-epidemiological possibilities, and Lars Melholt Rasmussen is an expert in biochemistry and biobanking, and as the head of the Elitary Research Centre CIMA, he will perform the independent administration of this complex project.

In addition, the members of the executive committee are assisted by the experts in the steering committee who will be responsible for securing local practical feasibility of the project at the specific screening sites. Meetings with the advisory board that consists of a multidisciplinary team of internationally recognised researchers, covering cardiovascular screening, health economics, advanced statistics, and translational cardiovascular medicine, will design and plan the optimal method of data-sampling including biosamples for scientific investigation. The design and plan will be implemented by the scientific committee, and finally, managed, analysed, and reported by the specific writing committees organised by the executive committee. (See the organisation chart for specific members and plan.)

Reference List

(1) Roger VL, Go AS, Lloyd-Jones DM, Adams RJ, Berry JD, Brown TM, et al. Heart disease and stroke statistics--2011 update: a report from the American Heart Association. Circulation 2011 Feb 1;123(4):e18-e209.

(2) http://www.hjerteforeningen.dk/film_og_boeger/udgivelser/hjertestatistik/. 2013.

(3) Sillesen H, Falk E. Why not screen for subclinical atherosclerosis? Lancet 2011 Aug 20;378(9792):645-6.

(4) Polonsky TS, Greenland P. CVD screening in low-risk, asymptomatic adults: clinical trials needed. Nat Rev Cardiol 2012 Oct;9(10):599-604.

(5) Lindholt JS, Sorensen J, Sogaard R, Henneberg EW. Long-term benefit and cost-effectiveness analysis of screening for abdominal aortic aneurysms from a randomized controlled trial. Br J Surg 2010 Jun;97(6):826-34.

(6) Simmons RK, Echouffo-Tcheugui JB, Sharp SJ, Sargeant LA, Williams KM, Prevost AT, et al. Screening for type 2 diabetes and population mortality over 10 years (ADDITION-Cambridge): a cluster-randomised controlled trial. Lancet 2012 Oct 3.

(7) Krogsboll LT, Jorgensen KJ, Gronhoj LC, Gotzsche PC. General health checks in adults for reducing morbidity and mortality from disease. Cochrane Database Syst Rev 2012;10:CD009009.

(8) Perk J, De BG, Gohlke H, Graham I, Reiner Z, Verschuren M, et al. European Guidelines on cardiovascular disease prevention in clinical practice (version 2012). The Fifth Joint Task Force of the European Society of Cardiology and Other Societies on Cardiovascular Disease Prevention in Clinical Practice (constituted by representatives of nine societies and by invited experts). Developed with the special contribution of the European Association for Cardiovascular Prevention & Rehabilitation (EACPR). Eur Heart J 2012 Jul;33(13):1635-701.

(9) Singh GM, Danaei G, Farzadfar F, Stevens GA, Woodward M, Wormser D, et al. The age-specific quantitative effects of metabolic risk factors on cardiovascular diseases and diabetes: a pooled analysis. PLoS One 2013;8(7):e65174.

(10) Diederichsen AC, Sand NP, Norgaard B, Lambrechtsen J, Jensen JM, Munkholm H, et al. Discrepancy between coronary artery calcium score and HeartScore in middle-aged Danes: the DanRisk study. Eur J Prev Cardiol 2012 Jun;19(3):558-64.

(11) Erbel R, Mohlenkamp S, Moebus S, Schmermund A, Lehmann N, Stang A, et al. Coronary risk stratification, discrimination, and reclassification improvement based on quantification of subclinical coronary atherosclerosis: the Heinz Nixdorf Recall study. J Am Coll Cardiol 2010 Oct 19;56(17):1397-406.

(12) Polonsky TS, McClelland RL, Jorgensen NW, Bild DE, Burke GL, Guerci AD, et al. Coronary artery calcium score and risk classification for coronary heart disease prediction. JAMA 2010 Apr 28;303(16):1610-6.

(13) Folsom AR, Kronmal RA, Detrano RC, O'Leary DH, Bild DE, Bluemke DA, et al. Coronary artery calcification compared with carotid intima-media thickness in the prediction of cardiovascular disease incidence: the Multi-Ethnic Study of Atherosclerosis (MESA). Arch Intern Med 2008 Jun 23;168(12):1333-9.

(14) Yeboah J, McClelland RL, Polonsky TS, Burke GL, Sibley CT, O'Leary D, et al. Comparison of novel risk markers for improvement in cardiovascular risk assessment in intermediate-risk individuals. JAMA 2012 Aug 22;308(8):788-95.

(15) Arad Y, Spadaro LA, Roth M, Newstein D, Guerci AD. Treatment of asymptomatic adults with elevated coronary calcium scores with atorvastatin, vitamin C, and vitamin E: the St. Francis Heart Study randomized clinical trial. J Am Coll Cardiol 2005 Jul 5;46(1):166-72.

(16) Houslay ES, Cowell SJ, Prescott RJ, Reid J, Burton J, Northridge DB, et al. Progressive coronary calcification despite intensive lipid-lowering treatment: a randomised controlled trial. Heart 2006 Sep;92(9):1207-12.

(17) Sogaard R, Laustsen J, Lindholt JS. Cost effectiveness of abdominal aortic aneurysm screening and rescreening in men in a modern context: evaluation of a hypothetical cohort using a decision analytical model. BMJ 2012;345:e4276.

(18) Criqui MH, Langer RD, Fronek A, Feigelson HS, Klauber MR, McCann TJ, et al. Mortality over a period of 10 years in patients with peripheral arterial disease. N Engl J Med 1992 Feb 6;326(6):381-6.

(19) Norgren L, Hiatt WR, Dormandy JA, Nehler MR, Harris KA, Fowkes FG, et al. Inter-Society Consensus for the Management of Peripheral Arterial Disease (TASC II). Eur J Vasc Endovasc Surg 2007;33 Suppl 1:S1-75.

(20) McDermott MM. The magnitude of the problem of peripheral arterial disease: epidemiology and clinical significance. Cleve Clin J Med 2006 Oct;73 Suppl 4:S2-S7.

(21) Wilson JMG, Jungner G. Principles and practice of screening for disease. Geneva. Geneva: WHO; 1968.

(22) Dupont WD, Plummer WD, Jr. Power and sample size calculations for studies involving linear regression. Control Clin Trials 1998 Dec;19(6):589-601.

(23) Moll FL, Powell JT, Fraedrich G, Verzini F, Haulon S, Waltham M, et al. Management of abdominal aortic aneurysms clinical practice guidelines of the European society for vascular surgery. Eur J Vasc Endovasc Surg 2011 Jan;41 Suppl 1:S1-S58.

(24) Mancia G, Fagard R, Narkiewicz K, Redon J, Zanchetti A, Bohm M, et al. 2013 ESH/ESC guidelines for the management of arterial hypertension: the Task Force for the Management of Arterial Hypertension of the European Society of Hypertension (ESH) and of the European Society of Cardiology (ESC). Eur Heart J 2013 Jul;34(28):2159-219.

(25) Ryden L, Grant PJ, Anker SD, Berne C, Cosentino F, Danchin N, et al. ESC Guidelines on diabetes, pre-diabetes, and cardiovascular diseases developed in collaboration with the EASD: the Task Force on diabetes, pre-diabetes, and cardiovascular diseases of the European Society of Cardiology (ESC) and developed in collaboration with the European Association for the Study of Diabetes (EASD). Eur Heart J 2013 Oct;34(39):3035-87.

(26) Lynge E, Sandegaard JL, Rebolj M. The Danish National Patient Register. Scand J Public Health 2011 Jul;39(7 Suppl):30-3.

(27) Helweg-Larsen K. The Danish Register of Causes of Death. Scand J Public Health 2011 Jul;39(7 Suppl):26-9.

(28) Pedersen CB. The Danish Civil Registration System. Scand J Public Health 2011 Jul;39(7 Suppl):22-5.

(29) Krarup LH, Boysen G, Janjua H, Prescott E, Truelsen T. Validity of stroke diagnoses in a National Register of Patients. Neuroepidemiology 2007;28(3):150-4.

(30) Nickelsen TN. [Data validity and coverage in the Danish National Health Registry. A literature review]. Ugeskr Laeger 2001 Dec 31;164(1):33-7.

(31) Nielsen HW, Tuchsen F, Jensen MV. [Validity of the diagnosis "essential hypertension" in the National Patient Registry]. Ugeskr Laeger 1996 Jan 8;158(2):163-7.

(32) Madsen M, Balling H, Eriksen LS. [The validity of the diagnosis of acute myocardial infarction in 2 registries: the Heart Registry compared to the National Patient Registry]. Ugeskr Laeger 1990 Jan 29;152(5):308-14.

(33) Preston DL, Ron E, Tokuoka S, Funamoto S, Nishi N, Soda M, et al. Solid cancer incidence in atomic bomb survivors: 1958-1998. Radiat Res 2007 Jul;168(1):1-64.

(34) Einstein AJ, Knuuti J. Cardiac imaging: does radiation matter? Eur Heart J 2012 Mar;33(5):573-8.

(35) Sattar N, Preiss D, Murray HM, Welsh P, Buckley BM, de Craen AJ, et al. Statins and risk of incident diabetes: a collaborative meta-analysis of randomised statin trials. Lancet 2010 Feb 27;375(9716):735-42.

(36) Baigent C, Blackwell L, Collins R, Emberson J, Godwin J, Peto R, et al. Aspirin in the primary and secondary prevention of vascular disease: collaborative meta-analysis of individual participant data from randomised trials. Lancet 2009 May 30;373(9678):1849-60.

(37) Lindholt JS, Juul S, Henneberg EW. High-risk and low-risk screening for abdominal aortic aneurysm both reduce aneurysm-related mortality. A stratified analysis from a single-centre randomised screening trial. Eur J Vasc Endovasc Surg 2007 Jul;34(1):53-8.

(38) Lindholt JS. Relatively high pulmonary and cardiovascular mortality rates in screening-detected aneurysmal patients without previous hospital admissions. Eur J Vasc Endovasc Surg 2007 Jan;33(1):94-9.

(39) Lindholt JS, Henneberg EW, Fasting H, Juul S. Mass or high-risk screening for abdominal aortic aneurysm. Br J Surg 1997 Jan;84(1):40-2.

Figure 1. Expected flow chart of the men included in the DANCAVAS trial

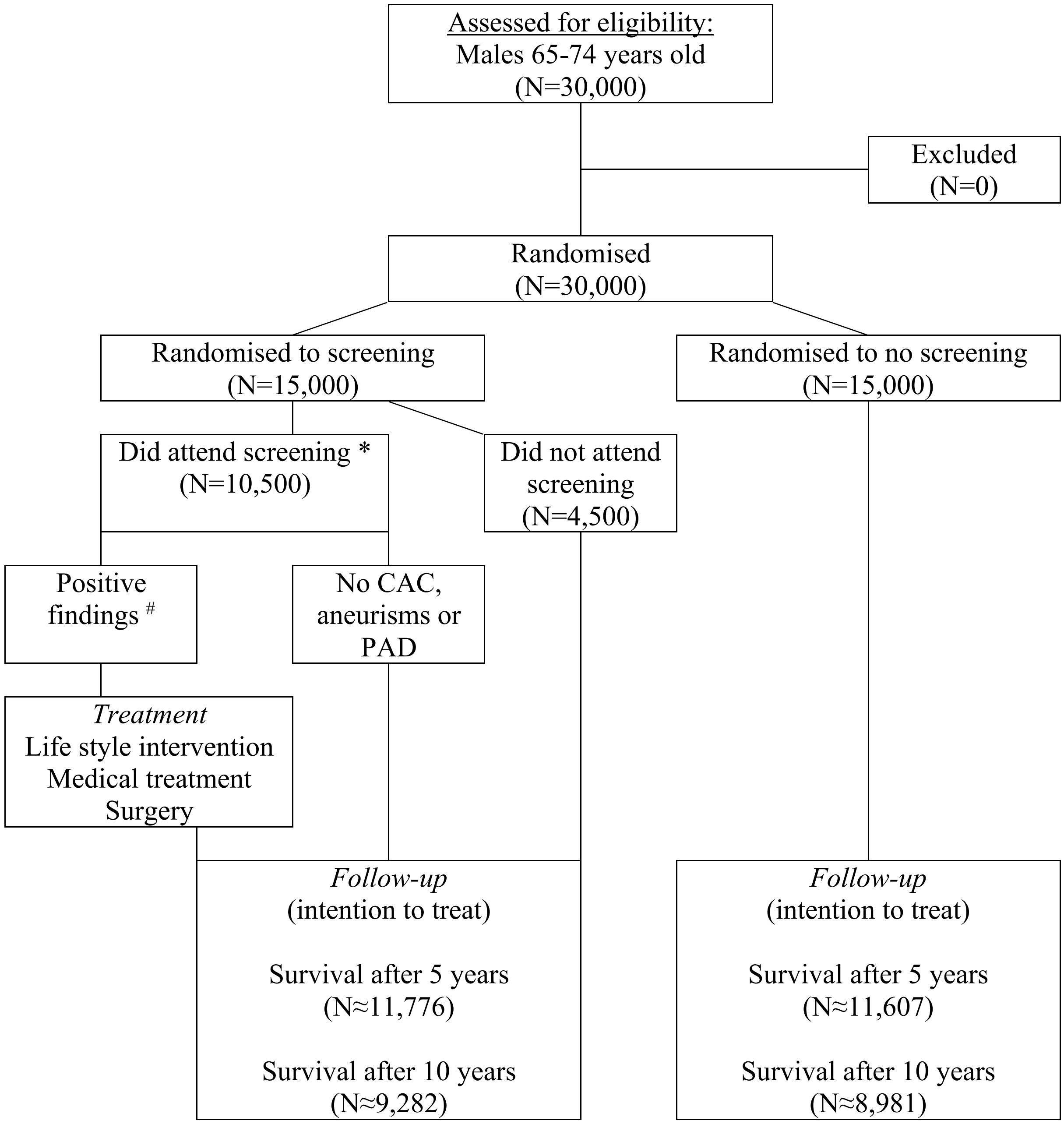

* Hypertension (systolic blood pressure >160 mmHg), diabetes mellitus (HbA1c >48 mmol/mol) or significant isolated hypercholesterolemia (total cholesterol >8.0 mmol/l) is suspected to be diagnosed in 1,470, 1,575, and 21 individuals, respectively. Further assessment and treatment will be left to the general practitioners.

# The screening is expected to find severe CAC, aneurysms and PADin 5,250, 525, and 1,050 patients, respectively.
