# Supplementary material for: Association of urate-lowering therapies with abdominal aortic aneurysm growth and clinical events in men: A population-based cohort study
Source: PLoS One. 2026 Jul 31;21(7):e0341242. doi: 10.1371/journal.pone.0341242 (PMC13427005; doi:10.1371/journal.pone.0341242)
Supplement: Attachment — Submitted filename: Response_to_reviewers (1).pdf [file pone.0341242.s007.pdf]

Dear Editor and Reviewers,

On behalf of our research team, we would like to thank you for your thorough evaluation of our study entitled Association of Urate-lowering Therapies with Abdominal Aortic Aneurysm Growth and Clinical Events in Men: A Population-Based Cohort Study

We sincerely appreciate the positive feedback and the opportunity to resubmit our revised manuscript.

The following includes our responses to the questions and remarks raised by The Editor, Reviewer #1 and Reviewer #2:

- Each question/remark is provided verbatim in **bold**, followed by our response (red).
- Sentences enclosed in quotation marks (" ") have been added or rephrased in the revised manuscript.
- Sentences written in *italic* indicate the section of the manuscript where the changes have been made.
- page and line numbers refer to the clean manuscript version.

We look forward to your assessment of our revision.

Yours sincerely and on behalf all authors,
Katja Bøhling Dybdahl Stud. MD., corresponding author and Mathilde Bernal, Stud MD.

Editor Remarks:

1. Please ensure that your manuscript meets PLOS ONE's style requirements, including those for file naming.

The manuscript has been revised to fully meet PLOS ONE's style requirements, including file naming.

2. Please note that funding information should not appear in any section or other areas of your manuscript. We will only publish funding information present in the Funding Statement section of the online submission form. Please remove any funding-related text from the manuscript.

Funding has now been deleted from the manuscript.

3. PLOS requires an ORCID iD for the corresponding author in Editorial Manager on papers submitted after December 6th, 2016. Please ensure that you have an ORCID iD and that it is validated in Editorial Manager. To do this, go to 'Update my Information' (in the upper left-hand corner of the main menu), and click on the Fetch/Validate link next to the ORCID field. This will take you to the ORCID site and allow you to create a new iD or authenticate a pre-existing iD in Editorial Manager.

ORCID iD for the corresponding author is now added ORCID iD: 0009-0009-7124-4671

4. One of the noted authors is a group or consortium: [DANCAVAS Trialists]. In addition to naming the author group, please list the individual authors and affiliations within this group in the acknowledgments section of your manuscript. Please also indicate clearly a lead author for this group along with a contact email address.

The requested is now added under Acknowledgements 402-419.

> "The authors acknowledge the contributions of the DANCAVAS trialists: Axel C. P. Diederichsen (Elite Centre for Individualized Medicine in Arterial Disease (CIMA), Odense University Hospital, Odense, Denmark and Department of Cardiology, Odense University Hospital, Odense, Denmark), Jes S. Lindholt (Department of Cardiothoracic and Vascular Surgery, Odense University Hospital, Odense, Denmark, Vascular Research Unit, University of Southern Denmark, Odense, Denmark, Elite Centre for Individualized Medicine in Arterial Disease (CIMA)), Flemming H. Steffensen (Department of Cardiology, Lillebaelt Hospital, Vejle, Denmark), Lars Frost (Department of Cardiology, Diagnostic Centre, Regional Hospital Silkeborg, Silkeborg, Denmark), Jess Lambrechtsen (Department of Medicine, Odense University Hospital, Svendborg, Denmark), Martin Busk (Department of Cardiology, Lillebaelt Hospital, Vejle, Denmark), Grazina Urbonaviciene (Department of Cardiology, Diagnostic Centre, Regional Hospital Silkeborg, Silkeborg, Denmark), Kenneth Egstrup (Department of Medicine, Odense University Hospital, Svendborg, Denmark), Marek Karon (Department of Medicine, Nykøbing Falster Hospital,

Nykøbing Falster, Denmark), and Lars M. Rasmussen (Elite Centre for Individualized Medicine in Arterial Disease (CIMA), Odense University Hospital, Odense, Denmark and Department of Clinical Biochemistry, Odense University Hospital, Odense, Denmark). The lead author of the group is Jes S Lindholt, contactable at jes.sanddal.lindholt@rsyd.dk"

5. Please include captions for your Supporting Information files at the end of your manuscript, and update any in-text citations to match accordingly.

This has now been incorporated at the end of the manuscript, and the in-text citations have been updated accordingly.

> **" S1 Table: Definitions**
> Table of definitions used, origin of data, and specific codes, measurements or timeframes applied in the study. ATC = Anatomical Therapeutic Chemical, ICD10 = International Statistical Classification of Diseases and Related Health Problems 10th Revision, SKS = The Danish Medical Classification System, DANCAVAS = The Danish Cardiovascular Screening trial, VIVA = The Viborg Vascular trial "

Corrected in-text citation on lines 180–181:

> "For a complete list of variable definitions, see the table in S1 Table."

> **"S2 File: VIVA ethics approval"**

Corrected in-text citation on lines 238-239:

> "The VIVA study was approved by the Ethics Committee of Central Denmark Region (journal number: M-20080028, see text in S2 File)..."

> **"S3 File: DANCAVAS ethics final approval "**
> **"S4 File: DANCAVAS ethics approval "**

Corrected in-text citation on lines 240-242:

> "The DANCAVAS trials were approved by the Southern Denmark Region Committee on Biomedical Research Ethics (journal number: S-20140028, see text in S3 File and S4 File) and the Danish Data Protection Agency. "

> **"S5 File: VIVA Study Protocol "**

> **"S6 File: DANCAVAS Study Protocol "**

6. Thank you for providing your underlying data as Supporting Information. We note that the data set contains text or data that is not in English. Please note that PLOS is an English-language publisher, so we require data sets to be provided in English as well. Please upload an English-language version of your data set.

Thank you for the reminder. The document uploaded on April 7th (S5_file.pdf) has now been completed with the previously missing sections in English.

Reviewer #1:

On a minor note, since Cox regression was used, please indicate that the proportional hazards assumption was, in fact, confirmed if such was the case.

The proportional hazards assumption was formally assessed for each Cox regression using scaled Schoenfeld residuals, globally and per covariate. The assumption was satisfied in the analyses of all-cause mortality, MACE, and the composite of surgery and rupture. In two of the three surgery-related outcomes, global tests indicated modest deviation (p between 0.01 and 0.04), attributable to baseline aortic size, with a time-interaction hazard ratio of 0.97 per year (95% CI 0.948 to 0.997), corresponding to approximately 14% attenuation over 5 years and an upper confidence limit close to 1. When stratified by study, the deviation was no longer significant within either subgroup, consistent with heterogeneity between imaging modalities (ultrasound and CT) rather than a true non-proportional effect. Primary estimates were materially unchanged in sensitivity analyses with a time-varying coefficient for aortic size, and the standard Cox model was retained.

The following has been added to the Methods and Results:

Methods line 219-222:

> "The proportional hazards assumption was assessed globally and for each covariate using scaled Schoenfeld residuals. Where deviations were observed, sensitivity analyses with time-varying coefficients were performed to confirm the robustness of the primary estimates."

Results Line 289-295:

> "The proportional hazards assumption was satisfied in the analyses of all-cause mortality, MACE, and the composite of surgery and rupture. In two of the three surgery-related outcomes, global Schoenfeld tests indicated modest deviation (p between 0.01 and 0.04), attributable to baseline aortic size (time-interaction hazard ratio 0.97 per year, 95% CI 0.948 to 0.997). The deviation was no longer significant when stratified by study, consistent with heterogeneity between imaging modalities. Primary estimates were materially unchanged in sensitivity analyses, including a time-varying coefficient for aortic size."

<u>Reviewer #2:</u>

Major feedback:

1) As authors themselves noted, the DANCAVAS and VIVA cohorts have notable heterogeneity in both the methods used and observed participant outcomes. Did the authors include study/measurement modality as covariate in their analyses and/or perform stratified analyses per cohort?

We have rephrased the covariates to improve clarity.

Method section line 195-202

> "The multivariable regressions were adjusted for the sub study, that includes the image modality and other well-established clinically and physiologically risk factors, including baseline age, baseline aorta size, body mass index (BMI), smoking status, diastolic blood pressure, diabetes, chronic obstructive pulmonary disease (COPD), chronic kidney disease, peripheral arterial disease, presence of other large vessel aneurysms, educational level and use of metformin, statins and urate-increasing drugs, including thiazides, loop diuretics and beta-blockers. "

Methods for secondary outcomes. line 214- 218.

> "The multivariable regressions were adjusted for the same covariates as those included in the primary outcome analyses. " *Given their relevance to clinical eligibility for surgery at the time, unoperated lung cancer and heart failure were also included in the adjusted models by the expert review of the senior author (JL).*

The legend for Table 3 has been clarified.

> "Adjusted for sub study, that includes the image modality, baseline age, baseline aorta size, body mass index, smoking status, diastolic blood pressure, diabetes, chronic obstructive pulmonary disease, chronic kidney disease, peripheral arterial disease, presence of other large vessel aneurysms, educational level, unoperated lung cancer, heart failure and use of metformin, statins and urate-increasing drugs, including thiazides, loop diuretics and beta-blockers. "

2) Please present data supporting the decision to model AAA growth rate with linear regression. Otherwise, were other models considered? Also clarify whether there were differences in the number and timing of scans between users and non-users.

Regarding the choice of linear regression to model AAA growth, this approach is consistent with previous large-scale studies, including publications from the DANCAVAS cohort, where AAA growth has been shown to approximate linear progression over time at the population level. In our dataset, visual inspection of growth distributions did not indicate

substantial deviations from linearity, thereby supporting the use of this model. Furthermore, evidence from a comparable AAA screening cohort of screening-detected cases followed using the same measurement methodology as in VIVA supports the assumption of approximately linear growth, with no clear deviations from linearity observed (Lindholt JS. *Abdominal aortic aneurysms*. Dan Med Bull. 2010 Dec;57(12):B4219, p. 27. PMID: 21122465).

Due to data protection regulations imposed by Statistics Denmark, DANCAVAS and VIVA were not permitted to upload individual-level longitudinal measurements to the analysis environment, as these may increase the risk of participant re-identification. Consequently, only aggregated data were available for analysis, and we therefore do not have access to detailed information on the number and timing of scans for users versus non-users. However, follow-up in both groups was conducted according to the same standardised surveillance protocols, and no systematic differences in scanning frequency between users and non-users are expected.

These data access limitations are briefly described in the first manuscript (lines 243–244): "Data from Statistics Denmark were anonymized, with counts under three (excluding zero) reported as '< 3' to protect participant privacy in accordance with regulations."

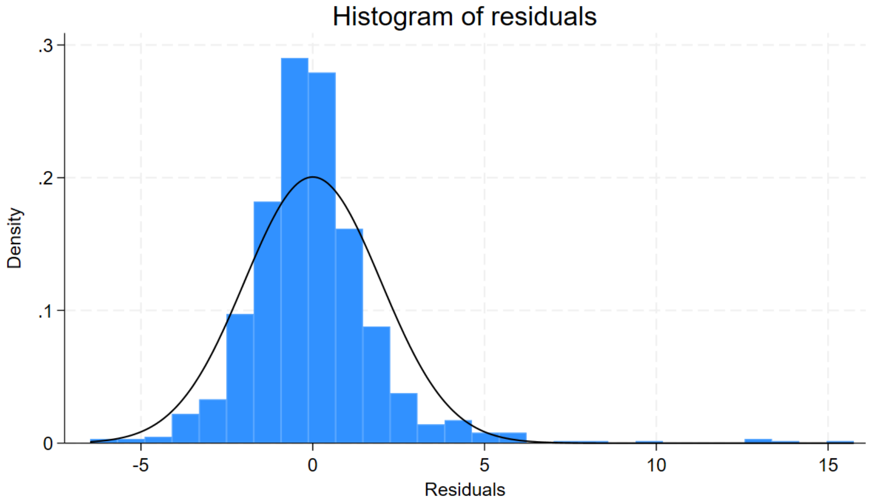

3) Was time from index date to first ULT exposure considered in the growth analyses? Could the straightforward averaged DDD approach dilute the effect in individuals who had shorter or intermittent exposure to ULT?

Time from index date to first ULT exposure was not explicitly accounted for in the growth analyses, as exposure was defined using averaged DDD over the follow-up period. As noted, this approach may dilute potential effects among individuals with delayed initiation or intermittent use.

However, as outlined in our previous responses, only aggregated data were available due to data protection regulations at Statistics Denmark. We therefore applied a pragmatic approach consistent with prior observational studies. Notably, a similar methodology has been used in a recent Circulation study of statin use and aneurysm progression, although statin exposure in that study was assigned within a trial setting. (Skovbo JS, Obel LM, Diederichsen ACP, Steffensen FH, Frost L, Lambrechtsen J, et al. Association of Statin Treatment and Dose With the Clinical Course of Small Abdominal Aortic Aneurysms in Men: A 5-Year Prospective Cohort Study From 2 Population-Based Screening Trials. Circulation.)

4) While the study is adequately powered to detect larger effects in growth rate, wide CIs suggests that more subtle effects can't be ruled out. Therefore, I'd suggest the authors to consider slightly tempering their ability to claim ULT's clinical significance.
We agree that the wide confidence intervals indicate that more modest effects cannot be excluded, and we have accordingly revised the conclusion to reflect this.

Added to the conclusion, line 392-394.

> "Although powered to detect clinically meaningful effects, smaller effects cannot be excluded and are unlikely to influence clinical practice."

Full conclusion:

> *In this population-based cohort study, urate-lowering therapies (ULT) use was not linked with a clinically significant reduction in AAA growth rate, risk of rupture, risk of needing AAA surgery, all-cause mortality or MACE over a five-year follow-up period.* "Although powered to detect clinically meaningful effects, smaller effects cannot be excluded and are unlikely to influence clinical practice." *ULT use was, however, associated with higher MACE occurrence. These results do not support the use of ULT to limit aneurysm progression. Further studies are needed to explore alternative pharmacologic treatments, including metformin, which show promise in ongoing trials, and statins, which have shown potential in a recent publication.*

Other minor feedback:
Check the consistent usage of ULT vs. urate-lowering therapy at first mention of each section.
According to your suggestion, we have corrected the use of ULT at first mention of each section to urate-lowering therapy.

Consider reiterating the definition and rationale of ULT at the beginning of the discussion.
We have now implemented your request in the beginning of the discussion, line 304-306.

> "Urate-lowering therapies (ULT), including xanthine oxidase inhibitors such as allopurinol and febuxostat, have been proposed to reduce oxidative stress and thereby potentially attenuate aneurysm progression."

While I appreciate the authors thorough study design, please consider streamlining Figure 1. as it is currently quite hard to follow. It would benefit greatly even from slight repositioning of elements to reduce overlap.

We agree that the figure can be challenging to follow due to the complexity of the subject. The figure has been revised and assessed multiple times, and we found that the current layout provides the clearest presentation of the information while maintaining all essential details.
