# Supplementary material for: Association of urate-lowering therapies with abdominal aortic aneurysm growth and clinical events in men: A population-based cohort study
Source: PLoS One. 2026 Jul 31;21(7):e0341242. doi: 10.1371/journal.pone.0341242 (PMC13427005; doi:10.1371/journal.pone.0341242)
Supplement: Peer review history

# Peer review: Decision Letter 0

**DOI:** 10.1371/journal.pone.0341242.r001

**Authors:** Xiang Zhu

13 Mar 2026

PONE-D-25-59482

Association of Urate-lowering Therapies with Abdominal Aortic Aneurysm Growth and Clinical Events in Men: A Population-Based Cohort Study

PLOS One

Dear Dr. Bøhling Dybdahl,

Thank you for submitting your manuscript to PLOS ONE. After careful consideration, we feel that it has merit but does not fully meet PLOS ONE’s publication criteria as it currently stands. Therefore, we invite you to submit a revised version of the manuscript that addresses the points raised during the review process.

Please submit your revised manuscript by Apr 27 2026 11:59PM. If you will need more time than this to complete your revisions, please reply to this message or contact the journal office at plosone@plos.org. When you're ready to submit your revision, log on to https://www.editorialmanager.com/pone/ and select the 'Submissions Needing Revision' folder to locate your manuscript file.

Please include the following items when submitting your revised manuscript:

- A letter that responds to each point raised by the academic editor and reviewer(s). You should upload this letter as a separate file labeled 'Response to Reviewers'.
- A marked-up copy of your manuscript that highlights changes made to the original version. You should upload this as a separate file labeled 'Revised Manuscript with Track Changes'.
- An unmarked version of your revised paper without tracked changes. You should upload this as a separate file labeled 'Manuscript'.

-->If you would like to make changes to your financial disclosure, please include your updated statement in your cover letter. Guidelines for resubmitting your figure files are available below the reviewer comments at the end of this letter.

If applicable, we recommend that you deposit your laboratory protocols in protocols.io to enhance the reproducibility of your results. Protocols.io assigns your protocol its own identifier (DOI) so that it can be cited independently in the future. For instructions see: https://journals.plos.org/plosone/s/submission-guidelines#loc-laboratory-protocols. Additionally, PLOS ONE offers an option for publishing peer-reviewed Lab Protocol articles, which describe protocols hosted on protocols.io. Read more information on sharing protocols at https://plos.org/protocols?utm_medium=editorial-email&utm_source=authorletters&utm_campaign=protocols.

We look forward to receiving your revised manuscript.

Kind regards,

Xiang Zhu

Academic Editor

PLOS One

Journal Requirements:

When submitting your revision, we need you to address these additional requirements.

1. Please ensure that your manuscript meets PLOS ONE's style requirements, including those for file naming. The PLOS ONE style templates can be found at

https://journals.plos.org/plosone/s/file?id=wjVg/PLOSOne_formatting_sample_main_body.pdf and https://journals.plos.org/plosone/s/file?id=ba62/PLOSOne_formatting_sample_title_authors_affiliations.pdf

2. Please note that funding information should not appear in any section or other areas of your manuscript. We will only publish funding information present in the Funding Statement section of the online submission form. Please remove any funding-related text from the manuscript.

3. PLOS requires an ORCID iD for the corresponding author in Editorial Manager on papers submitted after December 6th, 2016. Please ensure that you have an ORCID iD and that it is validated in Editorial Manager. To do this, go to ‘Update my Information’ (in the upper left-hand corner of the main menu), and click on the Fetch/Validate link next to the ORCID field. This will take you to the ORCID site and allow you to create a new iD or authenticate a pre-existing iD in Editorial Manager.

4. One of the noted authors is a group or consortium:

[DANCAVAS Trialists].

In addition to naming the author group, please list the individual authors and affiliations within this group in the acknowledgments section of your manuscript. Please also indicate clearly a lead author for this group along with a contact email address.

5. Please include captions for your Supporting Information files at the end of your manuscript, and update any in-text citations to match accordingly. Please see our Supporting Information guidelines for more information: http://journals.plos.org/plosone/s/supporting-information.

6. Thank you for providing your underlying data as Supporting Information.

We note that the data set contains text or data that is not in English. Please note that PLOS is an English-language publisher, so we require data sets to be provided in English as well. Please upload an English-language version of your data set.

This will also allow us to determine if your data follows PLOS standards per our Data Availability policy here: https://journals.plos.org/plosone/s/data-availability

If the reviewer comments include a recommendation to cite specific previously published works, please review and evaluate these publications to determine whether they are relevant and should be cited. There is no requirement to cite these works unless the editor has indicated otherwise.

Please review your reference list to ensure that it is complete and correct. If you have cited papers that have been retracted, please include the rationale for doing so in the manuscript text, or remove these references and replace them with relevant current references. Any changes to the reference list should be mentioned in the rebuttal letter that accompanies your revised manuscript. If you need to cite a retracted article, indicate the article’s retracted status in the References list and also include a citation and full reference for the retraction notice.

[Note: HTML markup is below. Please do not edit.]

Reviewers' comments:

Reviewer's Responses to Questions

**Comments to the Author**

1. Is the manuscript technically sound, and do the data support the conclusions?

The manuscript must describe a technically sound piece of scientific research with data that supports the conclusions. Experiments must have been conducted rigorously, with appropriate controls, replication, and sample sizes. The conclusions must be drawn appropriately based on the data presented.

Reviewer #1: Yes

Reviewer #2: Yes

**********

2. Has the statistical analysis been performed appropriately and rigorously?

Reviewer #1: Yes

Reviewer #2: Yes

**********

3. Have the authors made all data underlying the findings in their manuscript fully available?

The PLOS Data policy requires authors to make all data underlying the findings described in their manuscript fully available without restriction, with rare exception (please refer to the Data Availability Statement in the manuscript PDF file). The data should be provided as part of the manuscript or its supporting information, or deposited to a public repository. For example, in addition to summary statistics, the data points behind means, medians and variance measures should be available. If there are restrictions on publicly sharing data—e.g. participant privacy or use of data from a third party—those must be specified.

Reviewer #1: Yes

Reviewer #2: Yes

**********

4. Is the manuscript presented in an intelligible fashion and written in standard English?

PLOS ONE does not copyedit accepted manuscripts, so the language in submitted articles must be clear, correct, and unambiguous. Any typographical or grammatical errors should be corrected at revision, so please note any specific errors here.

Reviewer #1: Yes

Reviewer #2: Yes

**********

5. Review Comments to the Author

Please use the space provided to explain your answers to the questions above. You may also include additional comments for the author, including concerns about dual publication, research ethics, or publication ethics. (Please upload your review as an attachment if it exceeds 20,000 characters)

Reviewer #1: The design of this trial was very simple in that it utilized a cohort study with prospectively collected data from two large Danish population based screening trials. Sample size calculations were utilized indicating the least detectable tolerable difference which allowed for power considerations.

The statistical analysis was well outlined using both parametric and nonparametric for univariate continuous and categorical presentations. Linear regression was selected due to the linear pattern observed in the growth rates. The primary analysis compares AAA growth with the average daily use of ULT in defined daily dose (DDD) throughout the follow-up period. Incidence rates per 1 000 person-years were calculated for AAA rupture, risk of needing AAA surgery, all-cause mortality, and MACE, respectively. Unadjusted and adjusted Cox regression analyses were performed to assess the hazard ratios of ULT on these outcomes. Competing risks were wisely incorporated into the analysis as well.

The strengths and limitations were well noted. The authors did note that in some cases there was limited statistical power to detect smaller but potentially true associations, primarily due to a modest sample size and low ULT exposure (<1 DDD/day). Such limited exposure reduces the likelihood of observing a treatment effect.

The results followed from the analyses which were appropriate in that the use of urate-lowering therapies was not associated with a reduction in AAA progression. On a minor note, since Cox regression was used, please indicate that the proportional hazards assumption was, in fact, confirmed if such was the case.

Reviewer #2: This manuscript investigates whether urate-lowering therapies are associated with AAA growth rates and secondary outcomes in Danish men. The study is timely, well-motivated and relevant, as pharmacological treatment options for aneurysms remain limited to date. The authors show with rigorous statistical analyses that exposure to urate-lowering medications do not result in clinically significant reduction in observed AAA growth.

The study is most prominently limited in sample size due stringent censoring criteria and therefore also in statistical power. However, the work is overall well-conducted with thoughtfully implemented exposure modelling and does provide a concrete contribution to the field.

Major feedback:

1) As authors themselves noted, the DANCAVAS and VIVA cohorts have notable heterogeneity in both the methods used and observed participant outcomes. Did the authors include study/measurement modality as covariate in their analyses and/or perform stratified analyses per cohort?

2) Please present data supporting the decision to model AAA growth rate with linear regression. Otherwise, were other models considered? Also clarify whether there were differences in the number and timing of scans between users and non-users.

3) Was time from index date to first ULT exposure considered in the growth analyses? Could the straightforward averaged DDD approach dilute the effect in individuals who had shorter or intermittent exposure to ULT?

4) While the study is adequately powered to detect larger effects in growth rate, wide CIs suggests that more subtle effects can’t be ruled out. Therefore, I’d suggest the authors to consider slightly tempering their ability to claim ULT’s clinical significance.

Other minor feedback:

- Check the consistent usage of ULT vs. urate-lowering therapy at first mention of each section.

- Consider reiterating the definition and rationale of ULT at the beginning of the discussion.

- While I appreciate the authors thorough study design, please consider streamlining Figure 1. as it is currently quite hard to follow. It would benefit greatly even from slight repositioning of elements to reduce overlap.

**********

6. PLOS authors have the option to publish the peer review history of their article (what does this mean?). If published, this will include your full peer review and any attached files.

If you choose “no”, your identity will remain anonymous but your review may still be made public.

**Do you want your identity to be public for this peer review?**  For information about this choice, including consent withdrawal, please see our Privacy Policy.

Reviewer #1: No

Reviewer #2: No

**********

[NOTE: If reviewer comments were submitted as an attachment file, they will be attached to this email and accessible via the submission site. Please log into your account, locate the manuscript record, and check for the action link "View Attachments". If this link does not appear, there are no attachment files.]

To ensure your figures meet our technical requirements, please review our figure guidelines: https://journals.plos.org/plosone/s/figures

You may also use PLOS’s free figure tool, NAAS, to help you prepare publication quality figures: https://journals.plos.org/plosone/s/figures#loc-tools-for-figure-preparation.

NAAS will assess whether your figures meet our technical requirements by comparing each figure against our figure specifications.

# Author comment: Author response to Decision Letter 1

**DOI:** 10.1371/journal.pone.0341242.r002

15 May 2026

Reviewer #1:

On a minor note, since Cox regression was used, please indicate that the proportional hazards assumption was, in fact, confirmed if such was the case.

The proportional hazards assumption was formally assessed for each Cox regression using scaled Schoenfeld residuals, globally and per covariate. The assumption was satisfied in the analyses of all-cause mortality, MACE, and the composite of surgery and rupture. In two of the three surgery-related outcomes, global tests indicated modest deviation (p between 0.01 and 0.04), attributable to baseline aortic size, with a time-interaction hazard ratio of 0.97 per year (95% CI 0.948 to 0.997), corresponding to approximately 14% attenuation over 5 years and an upper confidence limit close to 1. When stratified by study, the deviation was no longer significant within either subgroup, consistent with heterogeneity between imaging modalities (ultrasound and CT) rather than a true non-proportional effect. Primary estimates were materially unchanged in sensitivity analyses with a time-varying coefficient for aortic size, and the standard Cox model was retained.

The following has been added to the Methods and Results:

Methods line 219-222:

"The proportional hazards assumption was assessed globally and for each covariate using scaled Schoenfeld residuals. Where deviations were observed, sensitivity analyses with time-varying coefficients were performed to confirm the robustness of the primary estimates."

Results Line 289-295:

"The proportional hazards assumption was satisfied in the analyses of all-cause mortality, MACE, and the composite of surgery and rupture. In two of the three surgery-related outcomes, global Schoenfeld tests indicated modest deviation (p between 0.01 and 0.04), attributable to baseline aortic size (time-interaction hazard ratio 0.97 per year, 95% CI 0.948 to 0.997). The deviation was no longer significant when stratified by study, consistent with heterogeneity between imaging modalities. Primary estimates were materially unchanged in sensitivity analyses, including a time-varying coefficient for aortic size."

Reviewer #2:

Major feedback:

1) As authors themselves noted, the DANCAVAS and VIVA cohorts have notable heterogeneity in both the methods used and observed participant outcomes. Did the authors include study/measurement modality as covariate in their analyses and/or perform stratified analyses per cohort?

We have rephrased the covariates to improve clarity.

Method section line 195-202

“The multivariable regressions were adjusted for the sub study, that includes the image modality and other well-established clinically and physiologically risk factors, including baseline age, baseline aorta size, body mass index (BMI), smoking status, diastolic blood pressure, diabetes, chronic obstructive pulmonary disease (COPD), chronic kidney disease, peripheral arterial disease, presence of other large vessel aneurysms, educational level and use of metformin, statins and urate-increasing drugs, including thiazides, loop diuretics and beta-blockers. “

Methods for secondary outcomes. line 214- 218.

“The multivariable regressions were adjusted for the same covariates as those included in the primary outcome analyses. “ Given their relevance to clinical eligibility for surgery at the time, unoperated lung cancer and heart failure were also included in the adjusted models by the expert review of the senior author (JL).

The legend for Table 3 has been clarified.

“Adjusted for sub study, that includes the image modality, baseline age, baseline aorta size, body mass index, smoking status, diastolic blood pressure, diabetes, chronic obstructive pulmonary disease, chronic kidney disease, peripheral arterial disease, presence of other large vessel aneurysms, educational level, unoperated lung cancer, heart failure and use of metformin, statins and urate-increasing drugs, including thiazides, loop diuretics and beta-blockers. “

2) Please present data supporting the decision to model AAA growth rate with linear regression. Otherwise, were other models considered? Also clarify whether there were differences in the number and timing of scans between users and non-users.

Regarding the choice of linear regression to model AAA growth, this approach is consistent with previous large-scale studies, including publications from the DANCAVAS cohort, where AAA growth has been shown to approximate linear progression over time at the population level. In our dataset, visual inspection of growth distributions did not indicate substantial deviations from linearity, thereby supporting the use of this model.

Furthermore, evidence from a comparable AAA screening cohort of screening-detected cases followed using the same measurement methodology as in VIVA supports the assumption of approximately linear growth, with no clear deviations from linearity observed (Lindholt JS. Abdominal aortic aneurysms. Dan Med Bull. 2010 Dec;57(12):B4219, p. 27. PMID: 21122465).

Due to data protection regulations imposed by Statistics Denmark, DANCAVAS and VIVA were not permitted to upload individual-level longitudinal measurements to the analysis environment, as these may increase the risk of participant re-identification. Consequently, only aggregated data were available for analysis, and we therefore do not have access to detailed information on the number and timing of scans for users versus non-users. However, follow-up in both groups was conducted according to the same standardised surveillance protocols, and no systematic differences in scanning frequency between users and non-users are expected.

These data access limitations are briefly described in the first manuscript (lines 243–244): “Data from Statistics Denmark were anonymized, with counts under three (excluding zero) reported as ‘< 3’ to protect participant privacy in accordance with regulations.”

(see histogram in the letter).

3) Was time from index date to first ULT exposure considered in the growth analyses? Could the straightforward averaged DDD approach dilute the effect in individuals who had shorter or intermittent exposure to ULT?

Time from index date to first ULT exposure was not explicitly accounted for in the growth analyses, as exposure was defined using averaged DDD over the follow-up period. As noted, this approach may dilute potential effects among individuals with delayed initiation or intermittent use.

However, as outlined in our previous responses, only aggregated data were available due to data protection regulations at Statistics Denmark. We therefore applied a pragmatic approach consistent with prior observational studies. Notably, a similar methodology has been used in a recent Circulation study of statin use and aneurysm progression, although statin exposure in that study was assigned within a trial setting. (Skovbo JS, Obel LM, Diederichsen ACP, Steffensen FH, Frost L, Lambrechtsen J, et al. Association of Statin Treatment and Dose With the Clinical Course of Small Abdominal Aortic Aneurysms in Men: A 5-Year Prospective Cohort Study From 2 Population-Based Screening Trials. Circulation.)

4) While the study is adequately powered to detect larger effects in growth rate, wide CIs suggests that more subtle effects can’t be ruled out. Therefore, I’d suggest the authors to consider slightly tempering their ability to claim ULT’s clinical significance.

We agree that the wide confidence intervals indicate that more modest effects cannot be excluded, and we have accordingly revised the conclusion to reflect this.

Added to the conclusion, line 392-394.

“Although powered to detect clinically meaningful effects, smaller effects cannot be excluded and are unlikely to influence clinical practice.”

Full conclusion:

In this population-based cohort study, urate-lowering therapies (ULT) use was not linked with a clinically significant reduction in AAA growth rate, risk of rupture, risk of needing AAA surgery, all-cause mortality or MACE over a five-year follow-up period. “Although powered to detect clinically meaningful effects, smaller effects cannot be excluded and are unlikely to influence clinical practice.” ULT use was, however, associated with higher MACE occurrence. These results do not support the use of ULT to limit aneurysm progression. Further studies are needed to explore alternative pharmacologic treatments, including metformin, which show promise in ongoing trials, and statins, which have shown potential in a recent publication.

Other minor feedback:

Check the consistent usage of ULT vs. urate-lowering therapy at first mention of each section.

- According to your suggestion, we have corrected the use of ULT at first mention of each section to urate-lowering therapy.

Consider reiterating the definition and rationale of ULT at the beginning of the discussion.

- We have now implemented your request in the beginning of the discussion, line 304-306.

“Urate-lowering therapies (ULT), including xanthine oxidase inhibitors such as allopurinol and febuxostat, have been proposed to reduce oxidative stress and thereby potentially attenuate aneurysm progression.”

While I appreciate the authors thorough study design, please consider streamlining Figure 1. as it is currently quite hard to follow. It would benefit greatly even from slight repositioning of elements to reduce overlap.

- We agree that the figure can be challenging to follow due to the complexity of the subject. The figure has been revised and assessed multiple times, and we found that the current layout provides the clearest presentation of the information while maintaining all essential details.

Attachment Submitted filename: Response_to_reviewers (1).pdf

# Peer review: Decision Letter 1

**DOI:** 10.1371/journal.pone.0341242.r003

**Authors:** Xiang Zhu

25 Jun 2026

<p>Association of Urate-lowering Therapies with Abdominal Aortic Aneurysm Growth and Clinical Events in Men: A Population-Based Cohort Study

PONE-D-25-59482R1

Dear Dr. Bøhling Dybdahl,

We’re pleased to inform you that your manuscript has been judged scientifically suitable for publication and will be formally accepted for publication once it meets all outstanding technical requirements.

Within one week, you’ll receive an e-mail detailing the required amendments. When these have been addressed, you’ll receive a formal acceptance letter and your manuscript will be scheduled for publication.

An invoice will be generated when your article is formally accepted. Please note, if your institution has a publishing partnership with PLOS and your article meets the relevant criteria, all or part of your publication costs will be covered. Please make sure your user information is up-to-date by logging into Editorial Manager at Editorial Manager® and clicking the ‘Update My Information' link at the top of the page. For questions related to billing, please contact billing support.

If your institution or institutions have a press office, please notify them about your upcoming paper to help maximize its impact. If they’ll be preparing press materials, please inform our press team as soon as possible -- no later than 48 hours after receiving the formal acceptance. Your manuscript will remain under strict press embargo until 2 pm Eastern Time on the date of publication. For more information, please contact onepress@plos.org.

Kind regards,

Xiang Zhu

Academic Editor

PLOS One

Additional Editor Comments (optional):

Reviewers' comments:

Reviewer's Responses to Questions

**Comments to the Author**

1. If the authors have adequately addressed your comments raised in a previous round of review and you feel that this manuscript is now acceptable for publication, you may indicate that here to bypass the “Comments to the Author” section, enter your conflict of interest statement in the “Confidential to Editor” section, and submit your "Accept" recommendation.

Reviewer #1: All comments have been addressed

Reviewer #2: (No Response)

**********

2. Is the manuscript technically sound, and do the data support the conclusions?

The manuscript must describe a technically sound piece of scientific research with data that supports the conclusions. Experiments must have been conducted rigorously, with appropriate controls, replication, and sample sizes. The conclusions must be drawn appropriately based on the data presented.

Reviewer #1: (No Response)

Reviewer #2: Yes

**********

3. Has the statistical analysis been performed appropriately and rigorously?

Reviewer #1: (No Response)

Reviewer #2: Yes

**********

4. Have the authors made all data underlying the findings in their manuscript fully available?

The PLOS Data policy requires authors to make all data underlying the findings described in their manuscript fully available without restriction, with rare exception (please refer to the Data Availability Statement in the manuscript PDF file). The data should be provided as part of the manuscript or its supporting information, or deposited to a public repository. For example, in addition to summary statistics, the data points behind means, medians and variance measures should be available. If there are restrictions on publicly sharing data—e.g. participant privacy or use of data from a third party—those must be specified.

Reviewer #1: (No Response)

Reviewer #2: Yes

**********

5. Is the manuscript presented in an intelligible fashion and written in standard English?

PLOS ONE does not copyedit accepted manuscripts, so the language in submitted articles must be clear, correct, and unambiguous. Any typographical or grammatical errors should be corrected at revision, so please note any specific errors here.

Reviewer #1: (No Response)

Reviewer #2: Yes

**********

6. Review Comments to the Author

Please use the space provided to explain your answers to the questions above. You may also include additional comments for the author, including concerns about dual publication, research ethics, or publication ethics. (Please upload your review as an attachment if it exceeds 20,000 characters)

Reviewer #1: All comments were addressed and revisions stated.

XXXXXXXXXXXXXXXXXXXXXXXXXXXXXXXXXXXXXXXXXXXXXXXXXXXXXXXXXXXXXXXXXXXXXXXXXXXXXXXXXXXXXX

Reviewer #2: The authors have addressed all of my methodological concerns adequately. I have no further revisions to propose.

While visualisation style is a subjective concern and I appreciate the authors' explanation that the current Figure 1. is the result of multiple iterations, I still believe the figure would have benefited from a redesign to make it more accessible to the broader readership of PLOS One. Given that the study design figure is often the element readers consult most, I believe clarity here would have meaningfully strengthened the paper.

**********

7. PLOS authors have the option to publish the peer review history of their article (what does this mean?). If published, this will include your full peer review and any attached files.

If you choose “no”, your identity will remain anonymous but your review may still be made public.

**Do you want your identity to be public for this peer review?**  For information about this choice, including consent withdrawal, please see our Privacy Policy.

Reviewer #1: No

Reviewer #2: No

**********

# Editor report: Acceptance letter

**DOI:** 10.1371/journal.pone.0341242.r004

**Authors:** Xiang Zhu

PONE-D-25-59482R1

PLOS One

Dear Dr. Bøhling Dybdahl,

I'm pleased to inform you that your manuscript has been deemed suitable for publication in PLOS One. Congratulations! Your manuscript is now being handed over to our production team.

At this stage, our production department will prepare your paper for publication. This includes ensuring the following:

* All references, tables, and figures are properly cited

* All relevant supporting information is included in the manuscript submission,

* There are no issues that prevent the paper from being properly typeset

You will receive further instructions from the production team, including instructions on how to review your proof when it is ready. Please keep in mind that we are working through a large volume of accepted articles, so please give us a few days to review your paper and let you know the next and final steps.

Lastly, if your institution or institutions have a press office, please let them know about your upcoming paper now to help maximize its impact. If they'll be preparing press materials, please inform our press team within the next 48 hours. Your manuscript will remain under strict press embargo until 2 pm Eastern Time on the date of publication. For more information, please contact onepress@plos.org.

You will receive an invoice from PLOS for your publication fee after your manuscript has reached the completed accept phase. If you receive an email requesting payment before acceptance or for any other service, this may be a phishing scheme. Learn how to identify phishing emails and protect your accounts at https://explore.plos.org/phishing.

If we can help with anything else, please email us at customercare@plos.org.

Thank you for submitting your work to PLOS ONE and supporting open access.

Kind regards,

PLOS ONE Editorial Office Staff

on behalf of

Dr. Xiang Zhu

Academic Editor

PLOS One
